# Effect of crystal-photodetector interface extraction efficiency on Cerenkov photons’ detection time

**DOI:** 10.3389/fphy.2022.1028293

**Published:** 2022-10-06

**Authors:** Carlotta Trigila, Emilie Roncali

**Affiliations:** 1 Department of Biomedical Engineering, University of California, Davis, Davis, CA, United States,; 2 Department of Radiology, University of California, Davis, Davis, CA, United States

**Keywords:** Cerenkov photons, index of refraction, transit time, detection time, detection efficiency

## Abstract

Using Cerenkov photons to improve detector timing resolution in time-of-flight positron emission tomography scanners is promising since they constitute most of the signal rising edge. The main challenge in using Cerenkov light is its low yield per photoelectric interaction, which requires optimizing their complex optical transport in the detector. Monte Carlo simulations unlock information unavailable through benchtop measurements and help better understand the Cerenkov photon behavior. Although the first Cerenkov photons are emitted forward, part of the early triggering signal is lost due to poor light extraction at the crystal-photodetector interface. In addition, the electron path in the crystal, that determines the Cerenkov photon direction, is tortuous due to multiple scattering, causing the Cerenkov photons emitted after a few scatters to no longer be forward-directed. In this context, the transit time spread in the crystal, highly dependent on the detector geometry, plays a crucial role in the photon detection time. In this work, we performed optical simulations in bismuth germanium oxide using 511 keV gamma with GATE to investigate the optical photons extraction when modifying the index of refraction at the crystal-photodetector interface and the crystal aspect ratio. The mean detection time of the first, second, and third detected optical and Cerenkov photon separately was studied as a function of the total number of Cerenkov detected per event. For each configuration, we calculated the expected mean detection time using the probability of detection. Thinner crystals led to lower expected detection times due to the reduced transit time in the crystal. Reducing the refractive index discontinuity at the crystal-photodetector interface decreased all configurations expected mean detection time values. We showed that it not only improves the optical photons (scintillation and Cerenkov) detection efficiency at the photodetector face but directly ameliorates the probability of detecting the fastest one, reducing the effect of thicker materials and of losing the first detected photon information, both crucial to reduce the detector timing resolution. Thanks to their prompt emission and directionality at emission, Cerenkov photons represent the first detected optical photon in most configurations but increasing their detection efficiency is crucial to detect the fastest one.

## 1 Introduction

The interest in employing time-of-flight Positron Emission Tomography (TOF-PET) systems has been renewed in recent years thanks to the emergence of fast scintillators, photodetectors, and electronics [1–6]. TOF-PET is effective at enhancing the images signal-to-noise ratio, executing faster image acquisitions, and thus reducing exposure rates for patients [7]. However, improving timing resolution in such systems demands understanding and optimizing all detector parameters influencing the timing resolution in the optical photon detection chain (crystal, photodetector, and readout electronics), and a common effort has been done in recent years by the community to address these problems.

Other groups have developed a comprehensive statistical model to predict the timing resolution of scintillation detectors [8]. The model, based on silicon photomultipliers (SiPMs), incorporated the relevant scintillator properties (i.e., the scintillation pulse shape, light yield) and SiPMs electronic response. Results showed that the optical photon counting statistics—determined by the average number of primary triggers, the scintillation rise time, decay time, and effective transit time spread—was an important and often dominant contributor to the overall timing performance of a scintillation detector.

Further improvement of radiation detectors timing will require to increase the optical photon detection efficiency, reduce the scintillator rise and decay times impact by using promptly emitted photons, enhance intrinsic light output through improved crystal growth and optimization of dopance and composition, optimize the light extraction and reduce transit time spread within the crystal. The latter comprises two major contributions. The first is the spread in the arrival time of the optical photons due to the variation of the gamma depth of interaction (DOI) in the crystal. The second contribution arises from the transit times of individual scintillation photons within the crystal (proportional to their path length), which is particularly sensitive to the optical properties of the material and the source energy—which both affect the photon directionality at emission-, to the material geometry, the reflector, and the coupling interfaces, and is particularly challenging to measure accurately.

In this context, Cerenkov photons’ prompt emission, combined with their predominantly forward emission direction with respect to the parent electron, makes them attractive for achieving superior timing resolution in TOF-PET [6, 10–13]. However, triggering gamma detections based on Cerenkov photons to improve radiation detector timing resolution is challenging due to the low number of photons produced per interaction (~17 from a 511 keV gamma in bismuth germanium oxide (BGO) [6]). To investigate the use of these prompt photons, accurately understanding their role compared to that of scintillation photons in the definition of detection time is of great interest since their detection time is dominated by their transit time in the crystal and the extraction efficiency at the photodetector face. Monte Carlo simulations are fundamental to model and study the Cerenkov detection pathway.

In previous works, we developed a high-fidelity model to perform accurate optical Monte Carlo simulations of radiation detectors, in which tracking individual optical photons relied on an accurate description of the reflections at the detector boundaries [14]. This validated model enables reliable optical simulation of radiation detectors within the well-validated high-energy physics simulation toolkit GATE [15]. We applied it to advanced optical Monte Carlo simulations of the transport and detection of Cerenkov photons in several materials [11, 17], optimizing the model to account for the Cerenkov photon emission properties [18, 19].

In this work, we investigated the effect of increasing the extraction efficiency and modifying the crystal’s geometry on scintillation and Cerenkov photons detection time in BGO. The index of refraction of the crystal-photodetector interface, material thickness, cross-section, and surface finish were varied. The photodetector and electronic responses were not included in this study. The optical photon mean detection time was studied as a function of the number of Cerenkov detected per gamma interaction for each configuration. We compared the results when considering all optical photons or only Cerenkov photons. Finally, we present the results in terms of expected mean detection time.

Due to the significant interest in understanding the effect of the crystal geometry and extraction efficiency at the photodetector face on optical photon detection and timing resolution, several studies have been performed on this topic [20–22]. To our knowledge, none of them focused on understanding the role of Cerenkov photons on the detection time and comparing them with the scintillation photons. Moreover, a complete and quantitative comparison of the effect of an increased extraction efficiency at the photodetector face has never been reported. The simulations presented in this work could be applicable to other radiation detector technologies with more complex detector designs, such as metacrystals [23].

## 2 Materials and methods

### 2.1 Simulation setup

Optical Monte Carlo simulations were performed in a BGO crystal with the toolkit GATE v9.0 (Geant4 10.06.03) [24]. BGO scintillation properties were defined as an isotropic emission following a single exponential decay with a time constant of 300 ns, a broad emission spectrum with a peak at 480 nm, and an index of refraction between 2.36 and 2.07 for wavelengths between 320 nm and 800 nm [25]. An optical absorption length between 13 and 2.5 cm was assumed in the same range [26]. The fast decay component of BGO (10% of the emission) and the rise time were not included. More details can be found in our previous work [11].

Four crystal thicknesses were sampled (each 5 mm from 5 mm to 20 mm), and three crystal cross-sections were used (2 × 2 mm^2^, 3 × 3 mm2, and 6 × 6 mm^2^). Two identical photodetectors were placed on opposite faces of the crystal in a dual-ended arrangement to improve the detector timing resolution [27]. Each photodetector was considered with an ideal photon detection efficiency of 1, including the geometrical and quantum efficiency. Since state-of-the-art PET detector uses a single-ended readout design, as comparison simulations were also performed using only one photodetector on the back face of the crystal.

Simulations were conducted using a 511 keV monoenergetic back-to-back source located 10 mm from the center of the BGO crystal perpendicular to the photodetector face. A total of 20,000 gamma photons were emitted per simulation, and only photoelectric interactions with the crystal were used in the simulations. The tortuous path of the energetic electron emitted by the gamma was modeled with a realistic inelastic mean free path in BGO at 511 keV in the order of a fraction of microns [19]. The optical photons produced by scintillation and Cerenkov emission from energetic electrons were simultaneously generated using the Livermore Model [28]. Optical photons transport was conducted using the LUT Davis model [15, 18]. Since scintillation and Cerenkov photons have different physical characteristics (emission spectrum and polarization), we generated and used separate LUTs according to the optical photon nature. We modified the Geant4/GATE source code to account for them simultaneously [18]. The crystal faces not in contact with the photodetector were all polished or rough, without a reflector or wrapped in a reflector (Teflon). The two faces in contact with the photodetectors were polished for all simulations. To model various crystal-photodetector couplings, we varied the index of refraction at the photodetector face from 1.5 to 2.2 with a step of 0.1. Values close to 1.5 are representative of the index of refraction of a commonly used optical grease or glue while 2.2 represents a value to minimize the mismatch with BGO.

**FIGURE 1:**
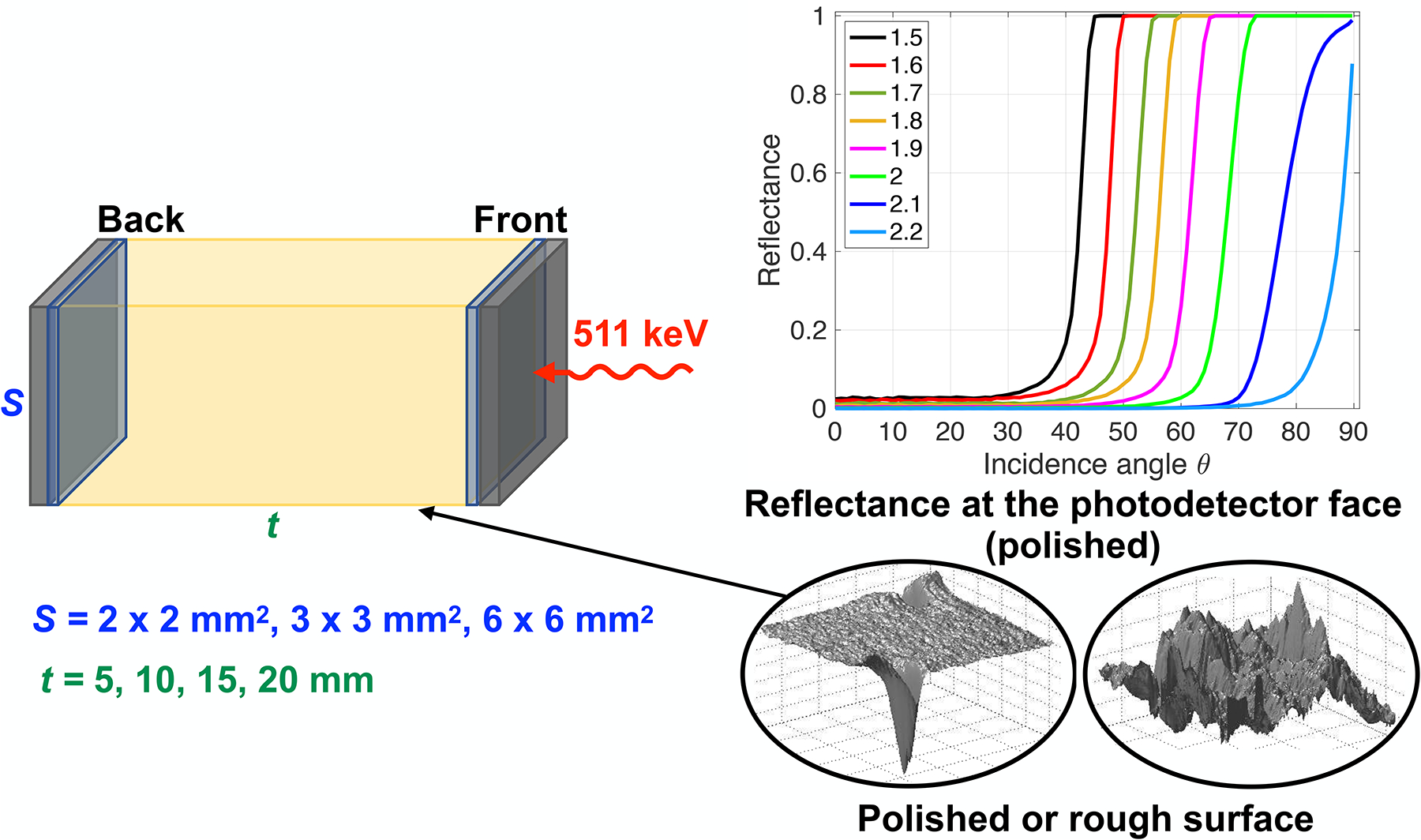
Schematic view of the simulated configurations. Left: Three cross-sections S and four material thicknesses t were modeled. Two identical photodetectors were placed on opposite faces of the crystal in a dual-ended arrangement. When a single-ended readout is used, only the Back photodetector was simulated. Bottom right: The crystal lateral edges were modeled as polished or rough, with or without a reflector (Teflon). Top right: The faces in contact with the photodetector were polished, and the index of refraction of the crystal-photodetector coupling was changed, which caused a changed reflectance at the photodetector face.

All simulated configurations are summarized in Figure 1.

### 2.2 Simulation analysis

For each configuration, the timing information of the gamma events and detected optical photons was saved in ROOT files [30]. The root output files were analyzed with MATLAB^®^ to reconstruct the complete history of each optical photon, identifying the parent gamma event and storing its nature (Scintillation or Cerenkov).

Each optical photon’s detection time was computed as the difference between its parent gamma entrance time at the front photodetector face and its arrival time on one of the two photodetectors. Consequently, the optical photon detection time included the gamma travel time within the crystal and the optical photon generation and transit time within the crystal. The generation time included the travel time of the recoil electron and, for the scintillation photons, their emission time due to the crystal decay time.

For each gamma interaction, we stored the discrete number of Cerenkov photons detected, the detection time of the first detected optical photon (scintillation or Cerenkov photon), and that of the first detected Cerenkov photon (not necessarily corresponding to the first detected optical photon). Their travel time and number of reflections before detection were also stored using a modified Geant4/GATE source code.

The gamma events were histogrammed according to the number of Cerenkov photons detected; the corresponding probability mass function of detected Cerenkov photons per gamma event was derived from the distribution. In addition, the mean detection time of the first optical photon (indistinctive of the photon nature), the mean detection time of the first Cerenkov photon, and the uncertainty of the detection time were calculated as a function of the number of Cerenkov photons detected. The uncertainties were estimated by the standard deviation.

The same study was performed using the detection time information of the second or third optical photon detected in an eventuality that the first or second optical photon arriving at the photodetector was not detected, respectively. This misdetection can result from a random deletion of a photon due to a low photodetection efficiency or a trigger on the signal, for example. The information of the first (or second) fast photon detected was removed from the sample of optical time stamps. If the deleted photon was emitted through the Cerenkov process, then the first Cerenkov detected information no longer corresponded to the first emitted Cerenkov photon. Alternatively, if the first optical photon was from scintillation, the first detected Cerenkov photons remained the same. These scenarios represent the extreme condition where all first or second optical photons arriving at the photodetector were lost and give a representative maximized description of a 50% or a 30% PDE on the simulated data, where not all first or second optical photons would be removed from the dataset.

For each configuration simulated, we first compared the first detected optical photon and first detected Cerenkov photon mean detection time values to highlight how the Cerenkov photon detection influences the detection time. Then, the results were expanded to the second and third detected optical photons. Last, we calculated the expected mean detection time of each configuration’s first, second, and third optical photon as the weighted average of the mean detection time as a function of the detected Cerenkov photons, where the probability mass function gave the weights, and we compared them.

## 3 Results

### 3.1 Crystal-photodetector optical coupling of 1.5

#### 3.1.1 Effect of the number of detected Cerenkov photons on optical photon timing

**FIGURE 2:**
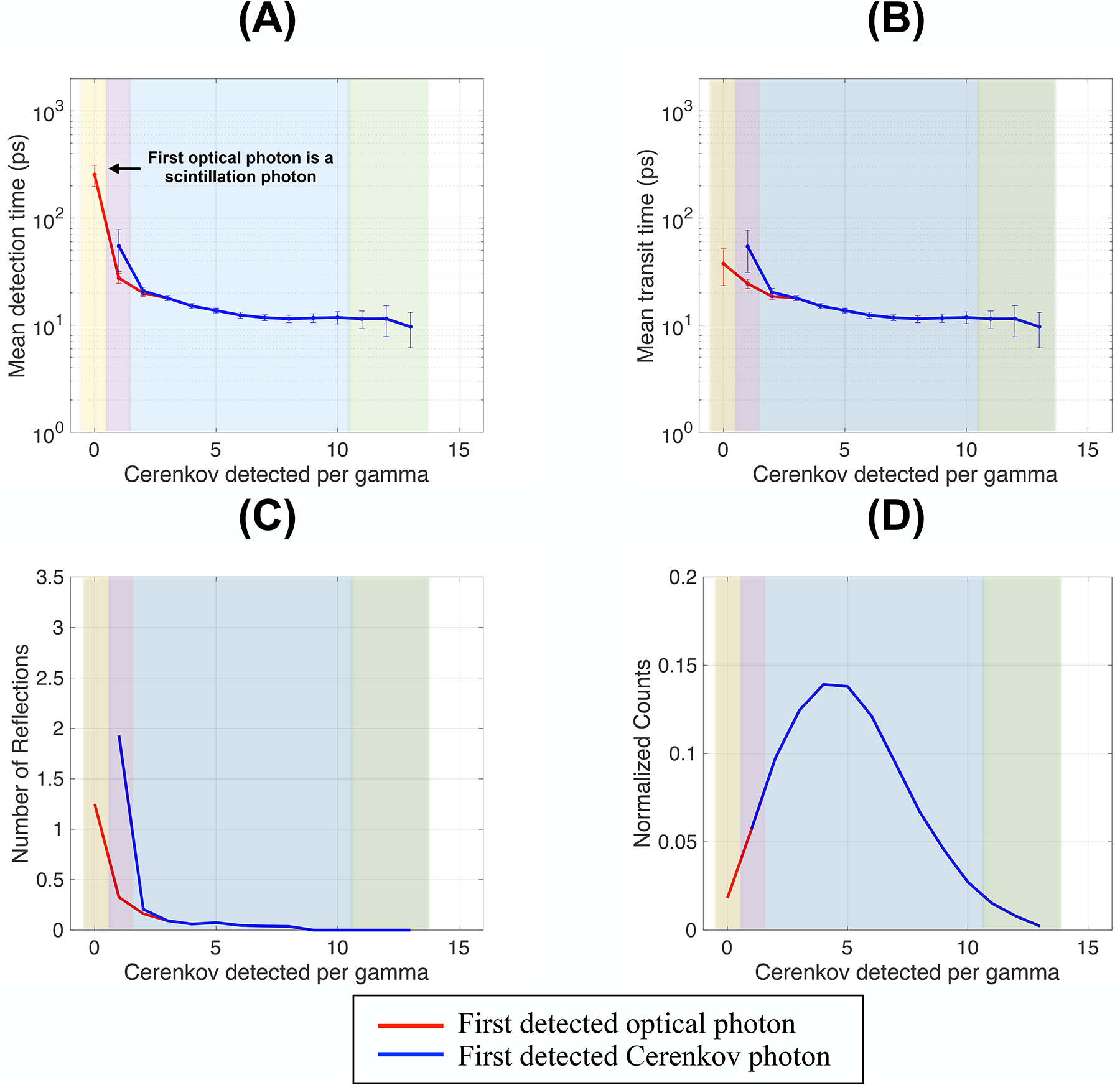
**(A)** Mean detection time, **(B)** travel time, **(C)** number of reflections, and **(D)** detected Cerenkov photons normalized distribution as a function of the Cerenkov photon detected per gamma. Results are shown for a 3 × 3 × 5 mm^3^ BGO crystal, with polished surfaces wrapped in a reflector (Teflon) and the photodetector faces coupled with an index of 1.5 (dual-ended readout arrangement).

Figure 2 shows the mean detection time, transit time, the number of reflections and the distribution of the number of detected Cerenkov photon per event of the first detected optical photon (in red) and of the first detected Cerenkov photon (in blue) as a function of the number of Cerenkov photons detected per gamma event. Results are shown for a 5 mm-thick polished 3 × 3 mm^2^ BGO crystal wrapped in Teflon and coupled to two photodetectors in dual-ended arrangement through a medium of refractive index 1.5. This configuration was used as a reference in the rest of the results.

With no Cerenkov photons detected (yellow regions in Figure 2), the contribution to the first detected optical photon mean detection time naturally came from scintillation photons only (first red dot in Figure 2A). The large mean detection time of ~300 ps was mainly due to the emission time following the slow BGO scintillation decay process. Indeed, among all isotropically emitted scintillation photons, the first detected ones experienced only few reflections (less than 1.5 on average, Figure 2C), which resulted in a transit time of only a few tens of ps (Figure 2B). The uncertainty in the mean detection time was due to the standard deviation of the scintillation photon detection time and the low frequency of gamma events with zero Cerenkov photon detected (Figure 2D).

As soon as at least one Cerenkov photon was detected (purple regions in Figure 2), the mean detection time of the first detected photon (second red dot in Figure 2A) decreased (~36 ps) thanks to the contribution of the detected Cerenkov photon. The first optical photon’s mean detection time was lower than that of the first detected Cerenkov photon (~62 ps, first blue dot in Figure 2A). This can be explained by considering that the probability that the unique detected Cerenkov photon represented the first emitted among all emitted Cerenkov photons (~17 per photoelectric interaction in BGO at 511 keV) was low. As opposed to the scintillation detection time, the Cerenkov detection time is primarily composed of the gamma travel time and its transit time in BGO, while its emission time is negligible. For the first emitted photon to be the first detected, the transit time must therefore be very short. The probability of this scenario is low, resulting in a large uncertainty on the mean detection time of this unique detected Cerenkov photon and a larger mean value than the mean scintillation detection time (purple regions in Figure 2).

When two or more Cerenkov photons were detected (blue and green regions in Figure 2), the mean detection time of the first optical photon detected was dominated by the Cerenkov photon contribution, as indicated by the overlap between the blue and red curves in Figure 2, meaning that the first optical photon detected was always a Cerenkov photon.

When the number of Cerenkov photons detected per gamma increased from two to seven (blue regions in Figure 2A), the mean detection time slightly decreased due to the increased probability of detecting the first emitted Cerenkov photons directed towards the photodetectors (reduced reflections, Figure 2C). Detecting four to five Cerenkov photons was the most frequent situation, as shown by the distribution peak in Figure 2D.

When the number of Cerenkov photons detected per gamma increased from seven to ten (green regions in Figure 2A), the mean detection time and travel time of the first photons detected did not change, reaching a constant plateau (~20 ps and ~11 ps, respectively). A plateau value of this order of magnitude can be justified by estimating the longest travel time of a directly detected optical photon (speed equal to c/n, with *n* = 2.1 at 480 nm) in a dual-ended arrangement when a gamma interacts in the middle of the crystal (d = 5 mm/2). This leads to a travel time of 17.5 ps. The lower value obtained (~11 ps) is the mean travel time considering different gamma DOIs in the material. The difference between the mean detection time and travel time plateau values (~9 ps) was due to the gamma travel time in the material (equal to a gamma traveling on average ~2.5 mm in BGO with *n* = 2.1).

These photons performed a very low number of reflections before detection (Figure 2C) and represented the fastest photon emitted and directly detected, resulting in reduced transit time spreads and, consequently, detection time uncertainties.

When more than nine Cerenkov photons were detected (green regions in Figure 2), the first detected photons were directly detected (zero reflections, Figure 2C). Although increasing the number of Cerenkov photons detected, the mean detection time of the first detected photons plateaued (~20 ps). However, the uncertainty increased (green regions in Figure 2) due to the decreased frequency of detection (Figure 2D) and the spread in the average arrival time of the optical photons due to the variation of the gamma position of interaction in the crystal, which could lead to very low detection time due to the detection of another Cerenkov photon emitted close to the photodetector face, which however does not affect the mean detection time.

When considering the same configuration in a single-ended readout (Supplementary Figure S1), the mean detection time, travel time, number of reflections, and distribution of detected Cerenkov photon per gamma trends were the same as described for the dual-ended readout. However, detecting only on one side of the crystal led to longer mean transit time values with larger uncertainties due to an increased optical photon travel time spread before detection (more reflections and longer path length to reach the single photodetector). The latter is also more sensitive to the gamma DOI within the crystal. The mean detection time consequently increased (~31 ps at the plateau).

#### 3.1.2 Effect of crystal geometry on optical photon timing

**FIGURE 3:**
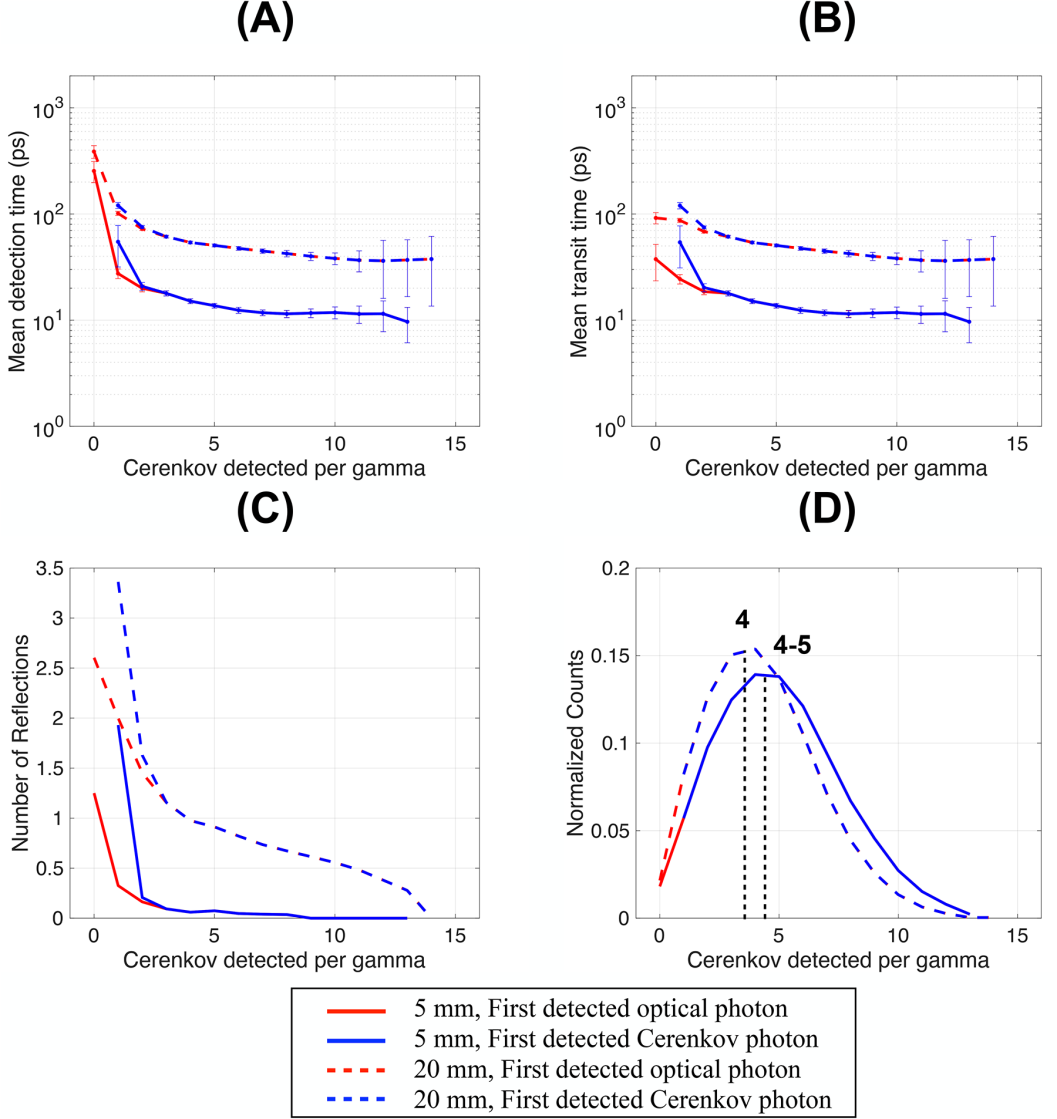
**(A)** Mean detection time, **(B)** travel time, **(C)** number of reflections, and **(D)** detected Cerenkov photons distribution as a function of the Cerenkov photon detected per gamma. Results are shown for a 3 × 3 × 20 mm^3^ and a 3 × 3 × 5 mm^3^ BGO crystal (same as Figure 2, repeated for clarity), with polished surfaces wrapped in a reflector (Teflon), and the photodetector faces coupled with an index of 1.5 (dual-ended readout arrangement).

Increasing the crystal thickness from 5 mm to 20 mm modified the optical photon distribution within the crystal. It increased the first detected optical photon transit time and number of reflections to reach the photodetector face and, consequently, their detection time and corresponding uncertainties (Figures 3A–C, dotted lines). It caused the detected Cerenkov photons normalized distribution to shift to a lower number of Cerenkov detected per gamma (Figure 3D, peak at 4 Cerenkov photons per gamma). It shows that thicker crystals led to a slightly reduced number of detected Cerenkov photons per gamma event for a given cross-section and surface finish. As already discussed for a 5 mm-thin crystal, the detection time with a thicker crystal also reached a plateau that weakly depended on the number of Cerenkov photons detected, and which uncertainties reflected the detected Cerenkov photons normalized distribution and spread in the average arrival time.

Modifying the crystal cross-section (2 × 2 mm^2^, 3 × 3 mm^2^, and 6 × 6 mm^2^) had a minimal effect on the mean detection time of the first optical photon and on the detected Cerenkov photons distribution (Supplementary Figure S2). With both thin and thick crystals, increasing the cross-section reduced the number of reflections of the first detected optical photon at the crystal lateral edges. This effect was more pronounced with thicker crystals, which have a smaller crystal aspect ratio. However, this did not reduce the transit time of the first detected optical photon since similar steps were needed to reach the photodetector face when larger cross-sections were used. In all these configurations (Supplementary Figure S2), as soon as two Cerenkov were detected per gamma event, they dominated the mean detection time.

**FIGURE 4:**
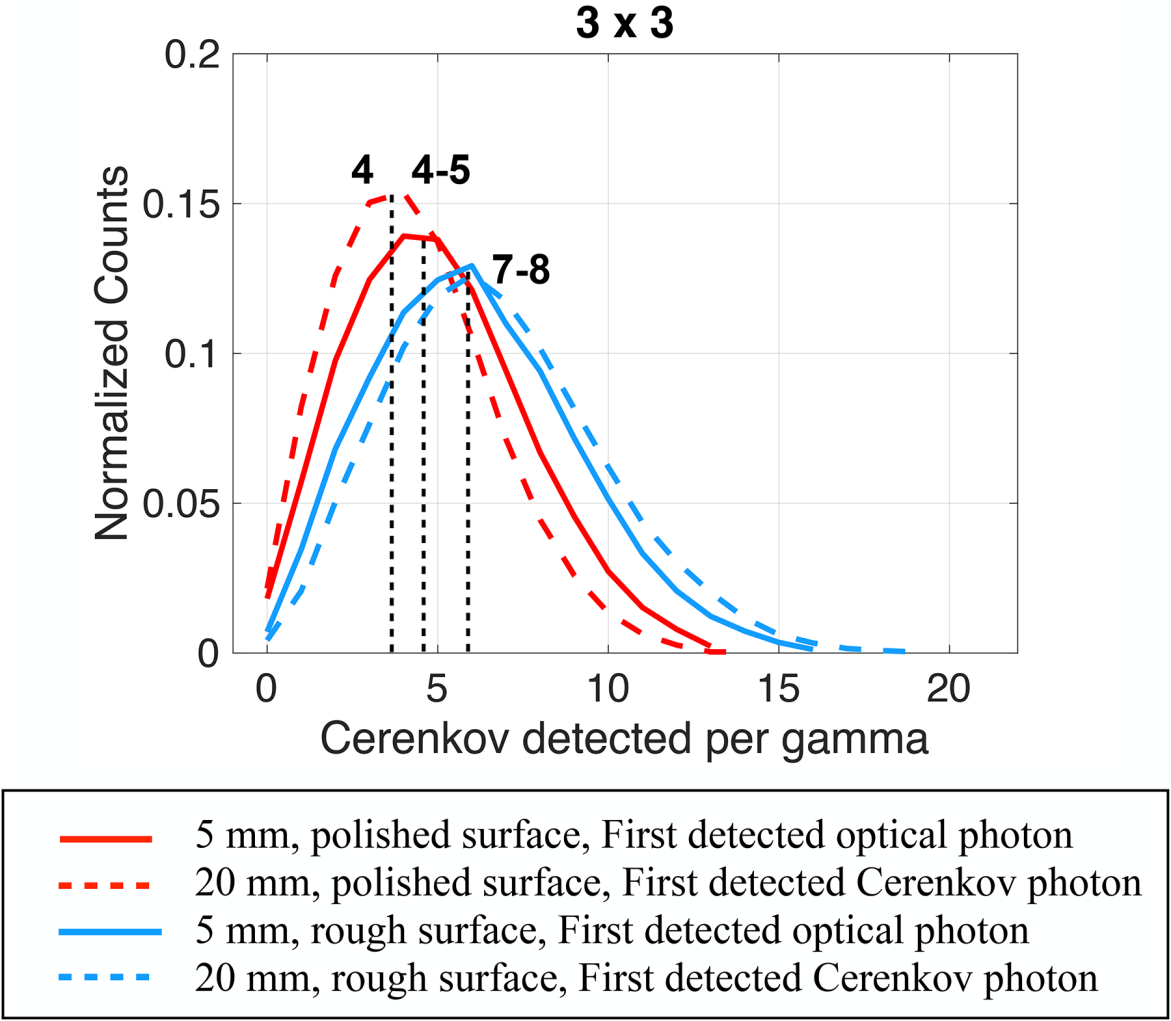
Detected Cerenkov photons distribution as a function of the Cerenkov photon detected per gamma. Results shown for a 3 × 3 mm^3^ BGO crystal, both 5 mm and 20 mm thick, with polished (red) and rough (light-blue) surfaces wrapped in a reflector (Teflon) and the photodetector faces coupled with an index of 1.5 (dual-ended readout arrangement).

When using rough surfaces wrapped in a Teflon reflector instead of polished ones, the mean detection time values as a function of the Cerenkov detected per gamma event slightly increased, mainly for thicker crystals and smaller cross-sections (not shown), where the first detected optical photons performed more reflections in their pathway to the photodetector. However, the rough surface allowed the detection of more Cerenkov photons per gamma event in all configurations, and the detected Cerenkov photons distribution peaks shifted to 7–8 Cerenkov detected per gamma event (Figure 4). The surface roughness changed the optical photon distribution within the crystal, causing slightly longer transit times but modified their angular distribution at the photodetector face leading to an increased extraction efficiency at the photodetector face.

Removing the reflector did not affect the detection time and the detected Cerenkov distribution of the first optical and Cerenkov photon when using a polished surface (not shown). However, removing the reflector on the rough surface reduced the mean detection time values as a function of the Cerenkov detected per gamma, mainly with thicker crystals and smaller cross-sections, but also reduced the number of Cerenkov photons detected per gamma event (Supplementary Figure S3). The effect is similar to what could be obtained when applying a black paint on crystal faces [31].

### 3.2 Increased crystal-photodetector optical coupling index of refraction

Increasing the crystal-photodetector coupling index of refraction reduced the index of refraction discontinuity at the crystal-photodetector interface (BGO has an index of refraction 2.15 at 480 nm and the photodetector has glass windows of ~1.5). It decreased the internal reflections at this interface, by reducing the total internal reflection range (reflectance curves in Figure 1), thus enhancing the extraction of the optical photons reaching the crystal-photodetector interface.

**FIGURE 5:**
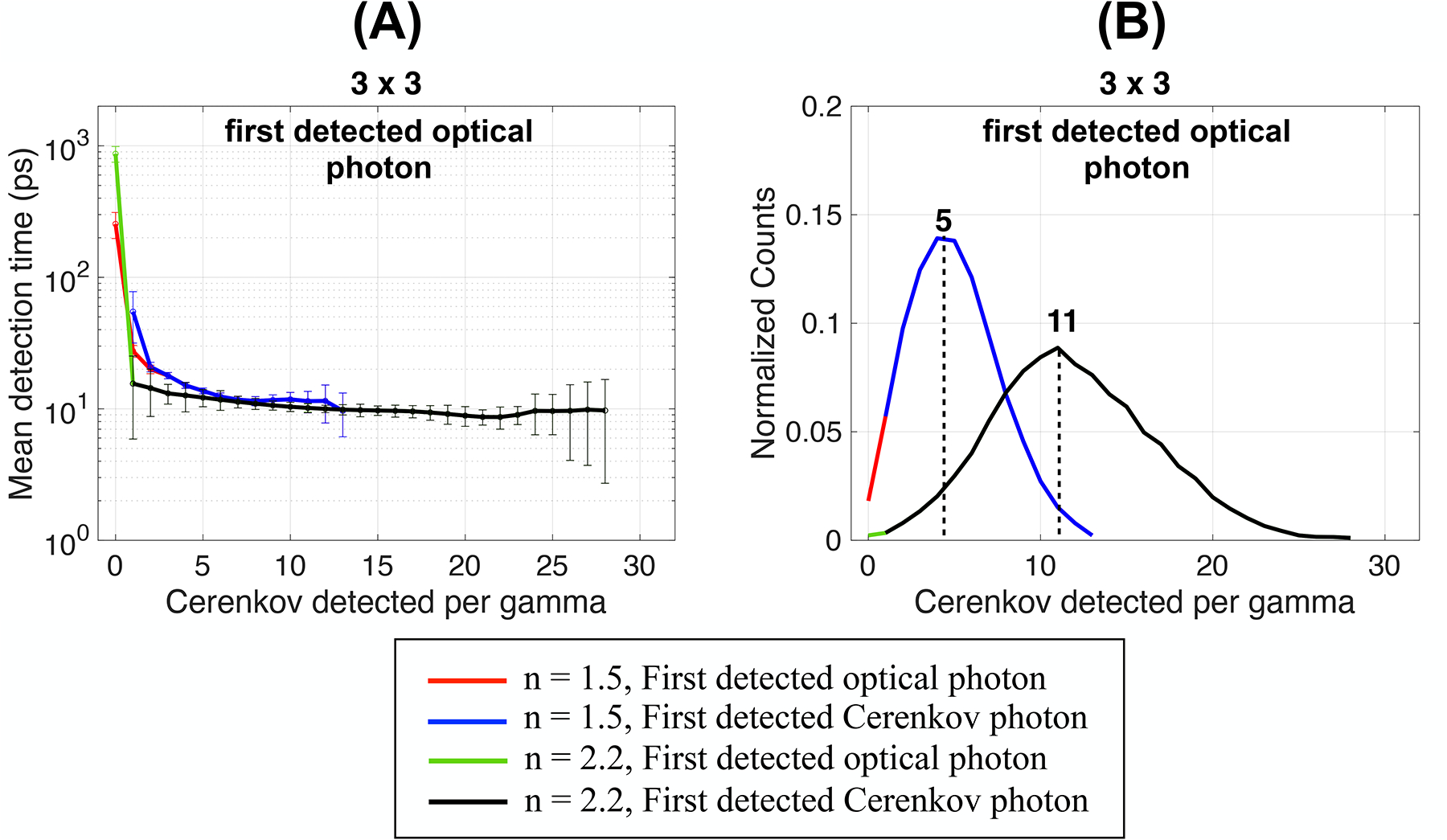
**(A)** Mean detection time, and **(B)** detected Cerenkov photons distribution as a function of the Cerenkov photon detected per gamma of the first detected optical and Cerenkov photons. Results are shown for a 3 × 3 × 5 mm^3^ BGO crystal, with polished surfaces wrapped in a reflector (Teflon), and the photodetector faces coupled with an index of 1.5 (same as Figure 2, repeated for clarity) or with an index of 2.2 (dual-ended readout arrangement).

With an index of 2.2, the mean detection time was dominated by the Cerenkov contribution (black and green curves overlap in Figure 5A), meaning that as soon as one Cerenkov photon was detected per gamma event, it always was the first optical photon detected (Figure 5A).

The mean detection time reached a plateau whose minimum value only slightly decreased compared to that obtained with a lower index of refraction (Figure 5A) since the first detected optical photons’ mean path within the crystal before detection did not change. However, the probability of extracting early Cerenkov increased, as shown by the increased maximum number of Cerenkov detected (28 instead of 13) and by the shift of the distribution peak (11 Cerenkov photons detected per gamma event instead of 5, Figure 5B). The larger uncertainties reflected the larger spread in the average arrival time of additional Cerenkov photons.

Similar behaviors to those described for an index of refraction of 1.5 (paragraph 3.2.1) were found with an index of 2.2 when increasing the crystal thickness, cross-section, and using a rough surface. When removing the reflector (Supplementary Figure S4), better detection times were obtained with the polished surface, while it worsened when using a rough surface (similarly to an index of 1.5, Supplementary Figure S2).

### 3.3 Second and third photon mean detection time trend

**FIGURE 6:**
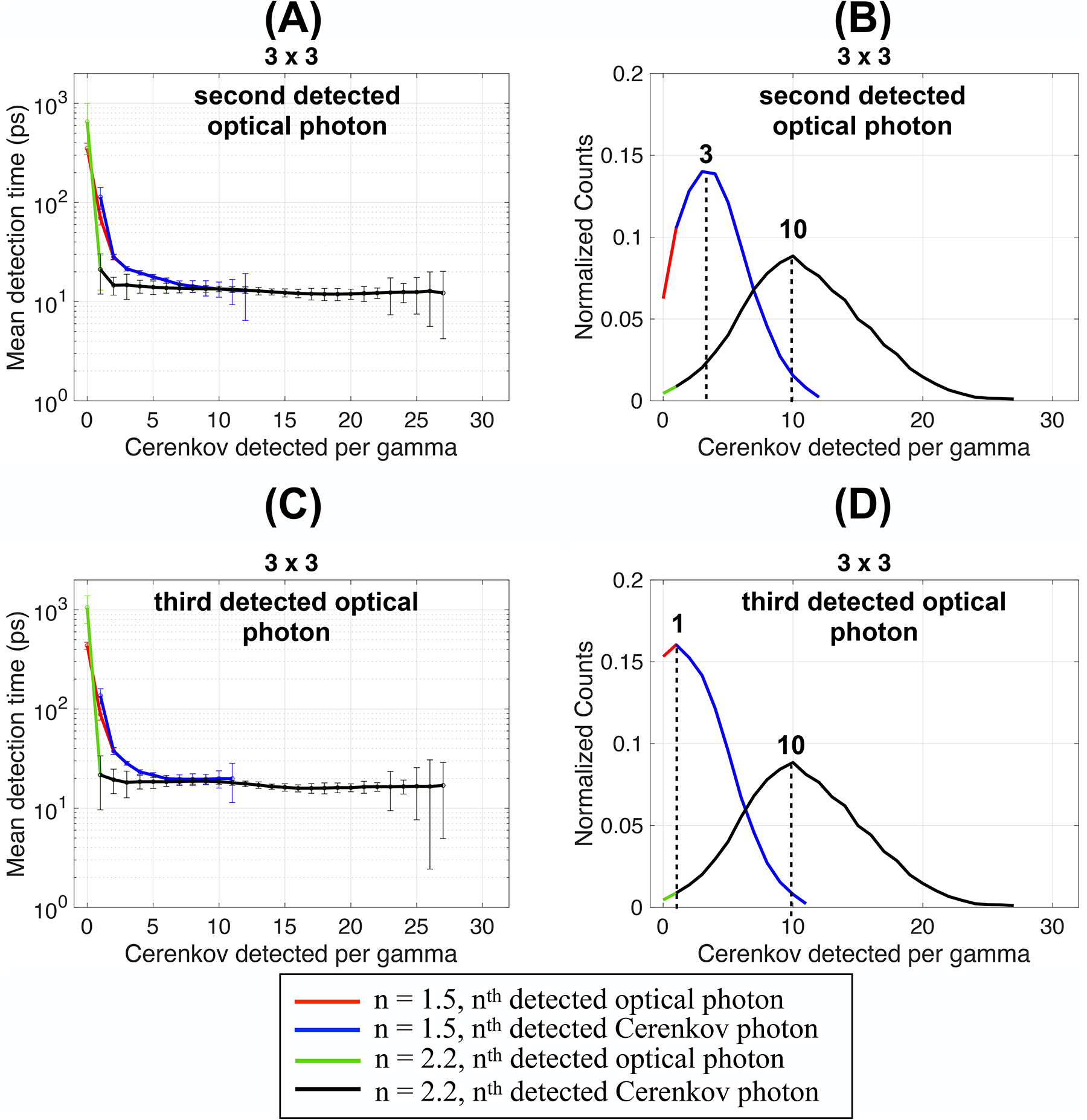
Mean detection time and detected Cerenkov photons distribution as a function of the Cerenkov photon detected per gamma calculated using the **(A,B)** second and **(C,D)** third detected optical photon and Cerenkov photon. Results are shown for a 3 × 3 × 5 mm^3^ BGO crystal, with polished surfaces wrapped in a reflector (Teflon) and the photodetector faces coupled with an index of 1.5 or 2.2 (dual-ended readout arrangement).

Using the detection time of the second (Figure 6A) and third (Figure 6C) detected optical photons slightly increased the mean detection times since they originated from slower photons. The detection time plateau increased from ~20 ps for the first detected optical photon to ~21 ps and ~24 ps for the second and third optical photon, respectively.

With the second detected photon, the distribution of the number of Cerenkov photons detected shifted to peaks at 3 and 10 for *n* = 1.5 and 2.2, respectively (Figure 6B). The peaks shifted to 1 and 10 for the third detected photon for *n* = 1.5 and 2.2, respectively (Figure 6D). For reference, when using the first detected optical photon the peaks were at 5 and 11 Cerenkov detected (Figure 5B). This indicates that the first three optical photons detected were often Cerenkov photons. With a coupling index of 2.2, the comparison between the mean time of all optical photons and Cerenkov photons shows that as soon as one Cerenkov photon was detected, it was the first optical photon detected, indicating its faster path to the photodetector.

## 4 Expected mean detection time

### 4.1 First optical photon

**FIGURE 7:**
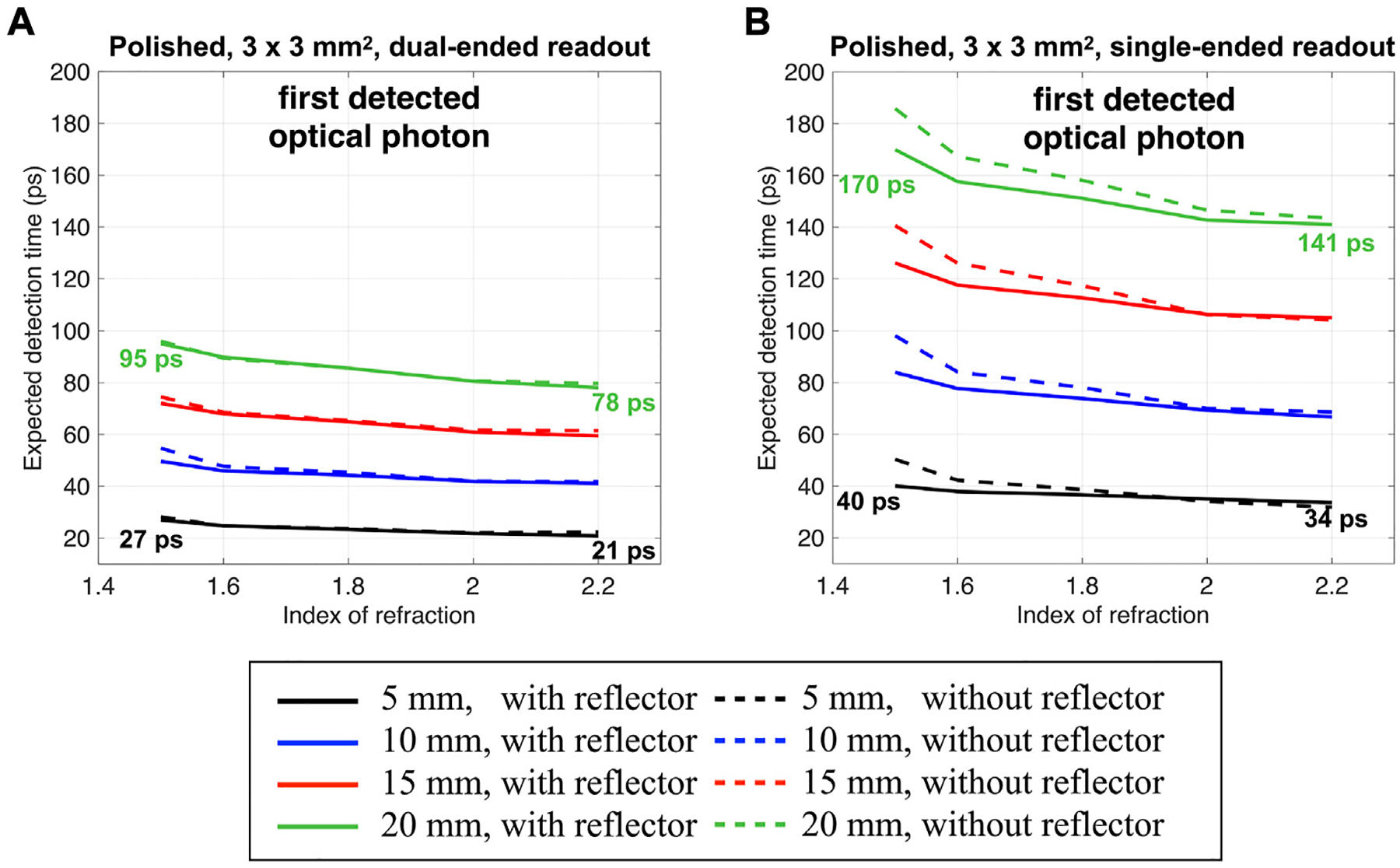
Expected detection time of the first detected optical photon as a function of the crystal-photodetector coupling index of refraction. Results are shown for a 3 × 3 mm^2^ cross-section, four thicknesses, polished surface on the crystal lateral edges with and without reflector, and for **(A)** a dual-ended readout or **(B)** a single-ended readout.

Figure 7 shows the expected mean detection time as a function of the crystal-photodetector coupling index of refraction for a 3 × 3 mm^2^ cross-section and four thicknesses (5 mm, 10 mm, 15 mm, and 20 mm). The expected mean detection time is the weighted average of the mean detection time using the distribution of detected Cerenkov photons per event as a probability mass function. Results are shown for the polish surface with reflector (bold lines) and without reflector (dotted lines) on the crystal lateral edges and using a dual-ended and single-ended readout. Results for other configurations are shown in Supplementary Figure S5.

When using a single-ended readout (Figure 7B), the detection time increase due to an increased optical photon travel time, and a stronger dependency on the gamma DOI (Supplementary Figure S1) led to an increased expected detection time for all configurations compared to the ones obtained with the dual-ended readout (Figure 7A).

The crystal thickness increased the expected detection time due to the increased transit time to reach the photodetector after emission (Figure 3). For example, a 27 ps expected detection time obtained with a 5 mm thick polished crystal raised to 95 ps when using a 20 mm thick material in a dual-ended photodetector arrangment (Figure 7A, index of refraction of 1.5). Same happened with the single-ended readout, where the expected detection time increased from 40 ps to 170 ps, obtained with a 5 mm and 20 mm thick polished crystal (Figure 7B, index of refraction of 1.5).

The expected mean detection time improved for all configurations when increasing the crystal-photodetector coupling index of refraction due to the higher probability of detecting early emitted Cerenkov photons (Figure 5B). For the dual-ended readout, for example, it led to a 22% and 18% expected detection time improvement for a 5 mm and a 20 mm thick polished material, respectively, when increasing the index of refraction from 1.5 to 2.2 (Figure 7A). For the same configurations, improvements of 15% and 17% were obtained when using the single-ended readout.

A decrease in the expected detection time as a function of the index of refraction was observed, although, for a given configuration, the mean detection time had the same minimum when increasing the index of refraction (Figure 5A). When using a lower index of refraction, larger weights were given to later events since the highest frequency was at ~5 events (Figure 5B blue curve). In contrast, with a larger index of refraction, larger weights were given to earlier events thanks to a higher number of detected photons (11 events, Figure 5B black curve). Although the higher index improved the expected detection time of the first detected optical photon, each crystal thickness showed the same trend, and the improvement was not sufficient to make up for the degradation due to the crystal thickness.

Since the rough surface increased the number of Cerenkov photons detected per gamma event (Figure 4), it led to a slightly improved expected detection time compared to the polished one when considering thinner materials and larger cross-sections (Supplementary Figure S5). The presence of a reflector had a limited influence on the first detected optical photon expected mean detection time when using a polished surface (dotted lines in Figure 7A). This was expected since no differences were found between the detection time and the detected Cerenkov distribution of the first optical and Cerenkov photon when using a polished surface and removing the reflector (as discussed in Section 3.1.2). With a rough surface (Supplementary Figure S5), removing the reflector had a larger effect, mainly when considering thick materials and smaller cross-sections. This is due to the strongly lowered probability of detecting Cerenkov photons when removing the reflector and increasing the thickness (Figure 4; Supplementary Figure S3, S4).

### 4.2 Second and third optical photon

**FIGURE 8:**
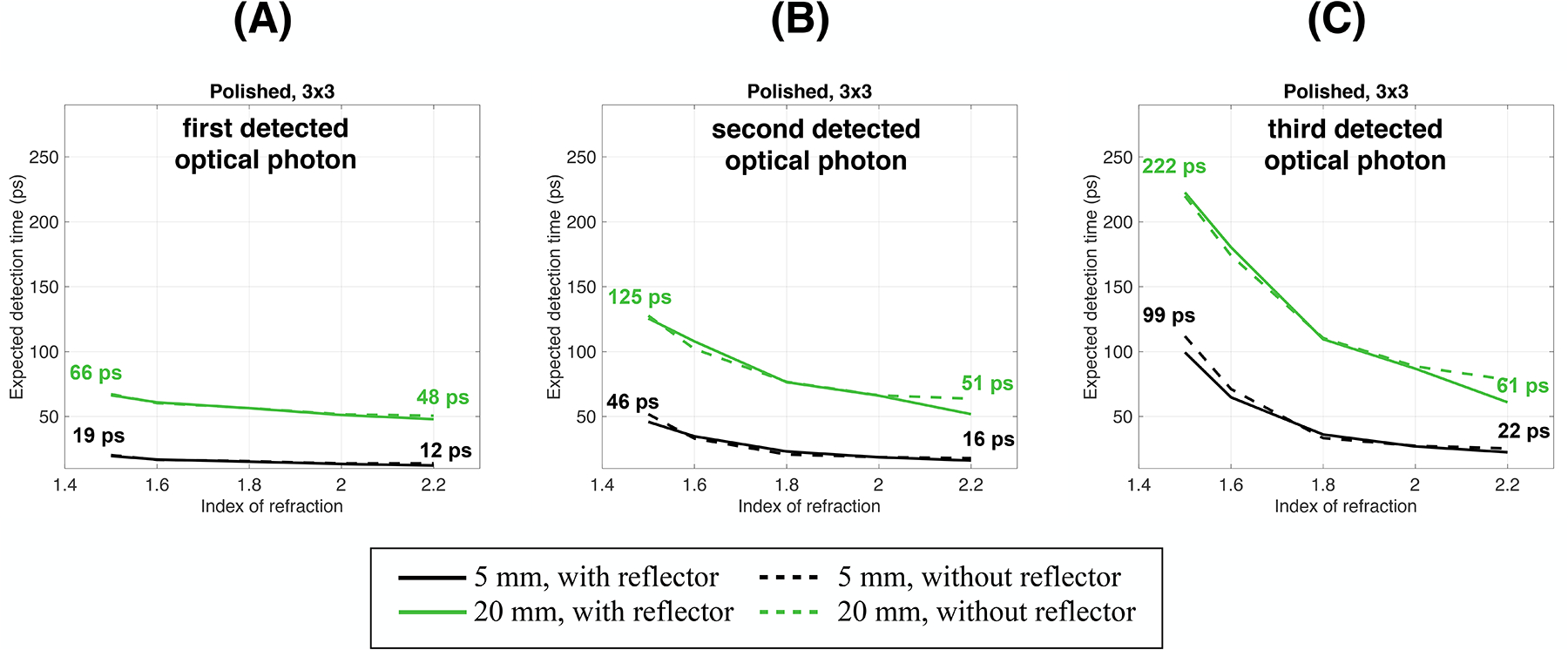
Expected detection time of the **(A)** first (same as Figure 7A, repeated for clarity), **(B)** second, and **(C)** third detected optical photon as a function of the crystal-photodetector coupling index of refraction. Results are shown for a 3 × 3 mm^2^ cross-section, two thicknesses, with and without a reflector, a polished surface on the crystal lateral edges and a dual-ended readout.

Losing the timing information of the first fastest optical photon reaching the photodetector worsened the expected detection time (Figure 8). With an index of refraction of 1.5, it almost doubled and quadrupled when considering the second and third optical photon detection time with respect to the first optical photon expected detection time (from 27 ps to 53 ps and 107 ps, Figure 8), and a 5 mm polished thick material. It increased by ~1.6 and ~2.6 times when a 20 mm thick material was considered (from 95 ps to 153 ps and 251 ps, Figure 8). This was mainly due to the important modification of the probability mass function distribution (Figures 5B, 6B), which is particularly important for all configurations where an already small number of Cerenkov photons were detected.

When increasing the index of refraction to 2.2, losing the information of the first or second optical photon detected moderately affected the expected mean detection time. It increased by ~1.1 and ~1.2 times with a 5 mm thick material (from 21 ps to 24 ps and 26 ps, Figure 8) and ~1.1 and ~1.3 times with a 20 mm thick material (from 78 ps to 91 ps and 105 ps, Figure 8).

It is important to note that with an index of 2.2, the expected detection time for the second or third photon (e.g., 24 ps and 26 ps at 5 mm, Figure 8) is close to or even better than that of the first photon expected detection time with an index of 1.5 (27 ps, Figure 8). This is true for both thicknesses.

Similar behaviors were obtained with the rough surface (Supplementary Figure S6). However, considering the second and third photons detected deteriorated less the expected detection time due to the larger probability of detecting faster Cerenkov photons obtained with the rough surface (Figure 4; Supplementary Figure S3).

Similar results were obtained using a single-ended readout (Supplementary Figure S7). Indeed, with a 5 mm thick material and an index of refraction of 1.5, the expected detection time almost doubled and quadrupled when considering the second and third optical photon detection time with respect to the first one (from 40 ps to 77 ps and 146 ps, Supplementary Figure S7). It increased by ~1.6 and ~2.5 times with a 20 mm thick (from 170 ps to 264 ps and 418 ps, Supplementary Figure S7). However, losing the information of the first or second optical photon detected less affected the expected mean detection time when increasing the index of refraction to 2.2. It increased by ~1.1 and ~1.3 times with a 5 mm thick material (from 34 ps to 39 ps and 46 ps, Figure 8) and ~1.1 and ~1.3 times with a 20 mm thick material (from 141 ps to 164 ps and 191 ps, Figure 8).

## 5 Discussion and conclusion

In this work, we investigated how using the prompt Cerenkov photons generated by the recoil electrons from the conversion of 511 keV gammas in BGO, together with an increased extraction efficiency at the crystal-photodetector face, could improve the detection time of crystal-based detectors.

The large refractive index mismatch at the crystal-photodetector interface, as the one existing between commonly used scintillator crystals (e.g., 2.15 BGO or 1.81 for LYSO) and optical grease (~1.5), represents the principal cause of an inefficient optical photon extraction in a radiation detector. Reducing the discontinuity between the crystal and the optical coupling indexes of refraction at the photodetector face represents a crucial point to improve the timing performance, when using a dual-ended or a single-ended readout. Reducing this discontinuity not only improves the optical (scintillation and Cerenkov) photon detection efficiency at the photodetector face but directly ameliorates the probability of detecting the fastest one, critical to reducing the detector timing resolution. It improves the information carried by the first detected optical photon since an increased probability of detecting more Cerenkov photons directly reduces the first Cerenkov photon detection time uncertainties and thus the resolution. Decreasing the index of refraction mismatch at the crystal-photodetector interface is even more important when losing the information of the first detected optical photon. For example, the expected detection time of the second optical photon detected was almost doubled when using a coupling index of refraction of 1.5, while it slightly changed with an index of 2.2.

We showed that thanks to their prompt emission, Cerenkov photons represent the first detected optical photon in most configurations, but that increasing their detection efficiency remains crucial to guarantee the detection of the fastest ones.

Since the light extraction also depends on the optical photon transit time and their angular distribution on the crystal-photodetector coupling interface, we investigated the detection time dependency on the crystal geometry (thickness and cross-section), surface finish, and reflector. The crystal thickness had a strong effect on the detection time. Not surprisingly, a thicker material led to a longer expected detection time due to a longer transit time within the crystal. Although shorter crystals yield faster time response, they generally lead to a significant loss of detector efficiency due to a reduced gamma attenuation in the thin material, and a good trade-off should be set with the aim of not compromising the detector efficiency while looking for the best timing resolution.

When able to use the first detected optical photon, although a higher index reduced the expected detection time, the degradation due to the transit time within the crystal remained the dominant contribution in thick crystals. However, when losing the information of the first detected optical photon and increasing the thickness, reducing the refractive index discontinuity at the crystal-photodetector interface remains crucial to assure the detection of the fastest ones. For example, we showed that the expected detection time of a 20 mm thick crystal coupled with a medium of index 2.2 led to a better expected detection time than that obtained with a 5 mm thick material coupled with grease (1.5) when losing the first and second optical photon.

We showed that using a dual-ended readout allowed reducing the optical photon mean detection time and uncertainties compared to a single-ended readout due to a reduced detection time dependence on the gamma DOI position and smaller travel time spread in the material. This can lead to better timing results, as already shown through experimental results [27]. However, since the single-ended readout still represents the state-of-the-art arrangement used in TOF-PET, interesting to highlight is that, as happens with the dual-ended readout, reducing the refractive index discontinuity at the crystal-photodetector interface improves the optical photons (scintillation and Cerenkov) detection efficiency at the photodetector face and directly ameliorates the probability of detecting the fastest one, reducing the effect of thicker materials and of losing the first detected photon information, both crucial to reduce the detector timing resolution.

One possible solution to improve TOF-PET timing resolution using prompt Cerenkov photons while reducing the crystal-photodetector coupling mismatch is the direct integration of a Cerenkov radiator (e.g., Lead fluoride glass PbF2, lead-tungstate crystals PbW04) with the photocathode inside a microchannel plate photomultiplier tube (MCP-PMTs) [32]. Although extremely promising in terms of timing resolution, which demonstrated the possibility of reconstruction-free imaging [33], the absence of scintillation photons in these pure Cerenkov emitters led to low detection sensitivity, making them not suitable for system TOF-PET at their current state-of-the-art.

A promising solution to address the refractive index discontinuity problem using scintillator-based radiation detectors and consequently mitigate the effect of using longer crystals is to enhance the optical photon extraction efficiency at the crystal photodetector interface by using photonic crystals (PhCs). A photonic crystal is a thin layer of dielectric material with a large index of refraction (e.g., Titanium dioxide TiO2, 2.1–2.4 index of refraction) imprinted on the scintillator with a periodic nanostructure [34]. If the periodicity is of the order of the wavelength of the light, they have the potential to significantly enhance light extraction [35–37].

The potential of this technique is far from being exhausted and modeling these emerging technologies must be enabled in current optical simulation capabilities. Using periodic nanostructures, PhCs need to be described with wave optics, which is incompatible with the geometric optics and particle tracking implemented in Monte Carlo simulators like GATE/Geant4. We are developing a novel hybrid wave-particle optics model of these complex interfaces that will be integrated into the LUT Davis model after experimental validation. It will allow researchers to study custom crystal-PhC-photodetector assemblies in GATE for the first time.

## Supplementary Material

Figure S3

Figure S1

Figure S2

Figure S4

Figure S5

Figure S6

Figure S7

Figure S8

Figure S9

Figure S10

## Data Availability

The raw data supporting the conclusion of this article will be made available by the authors, without undue reservation.
