# Supplementary material for: Effect of crystal-photodetector interface extraction efficiency on Cerenkov photons’ detection time
Source: Front Phys. Author manuscript; Available in PMC 2024 Dec 23. (PMC11666256; doi:10.3389/fphy.2022.1028293)
Supplement: Figure S3 [file NIHMS2002029-supplement-Figure_S3.pdf]

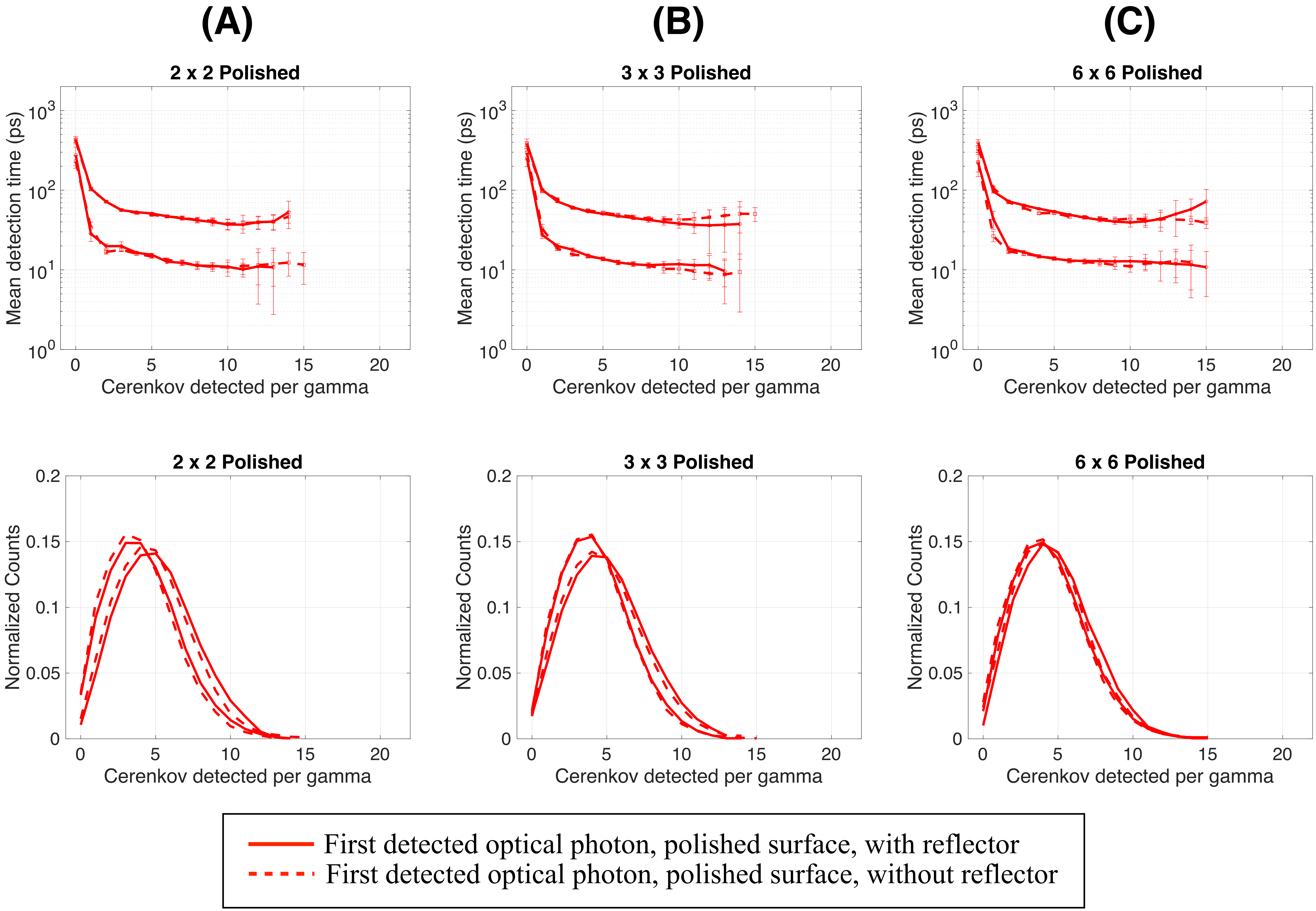

(A)
(B)
(C)
2 x 2 Polished
3 x 3 Polished
6 x 6 Polished
Mean detection time (ps)
10^3
10^2
10^1
10^0
Normalized Counts
0.2
0.15
0.1
0.05
0
0
5
10
15
20
Cerenkov detected per gamma
First detected optical photon, polished surface, with reflector
First detected optical photon, polished surface, without reflector
