# Supplementary figures and images for: Effect of crystal-photodetector interface extraction efficiency on Cerenkov photons’ detection time
Source: Front Phys. Author manuscript; Available in PMC 2024 Dec 23. (PMC11666256; doi:10.3389/fphy.2022.1028293)

**(A)****2 x 2**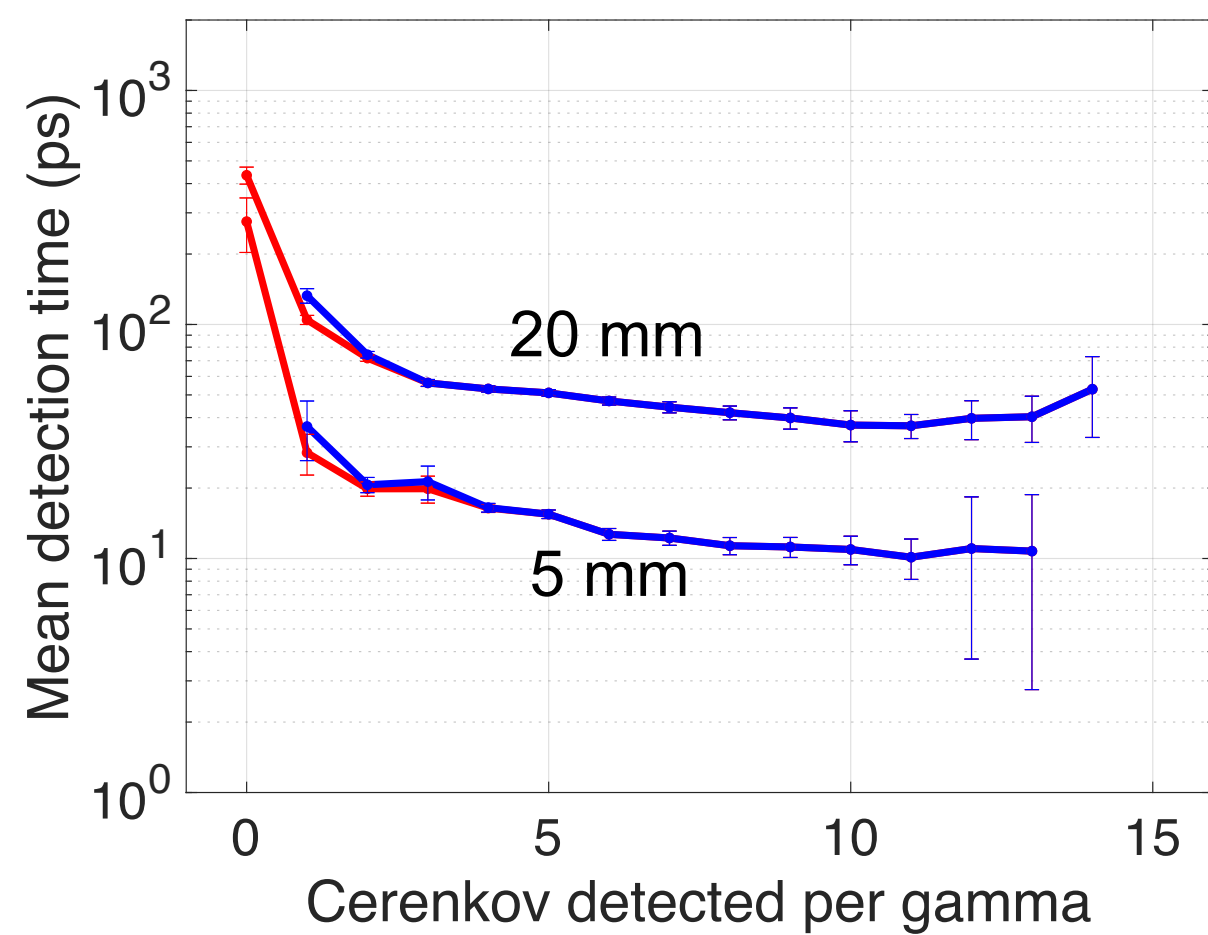**(B)****3 x 3**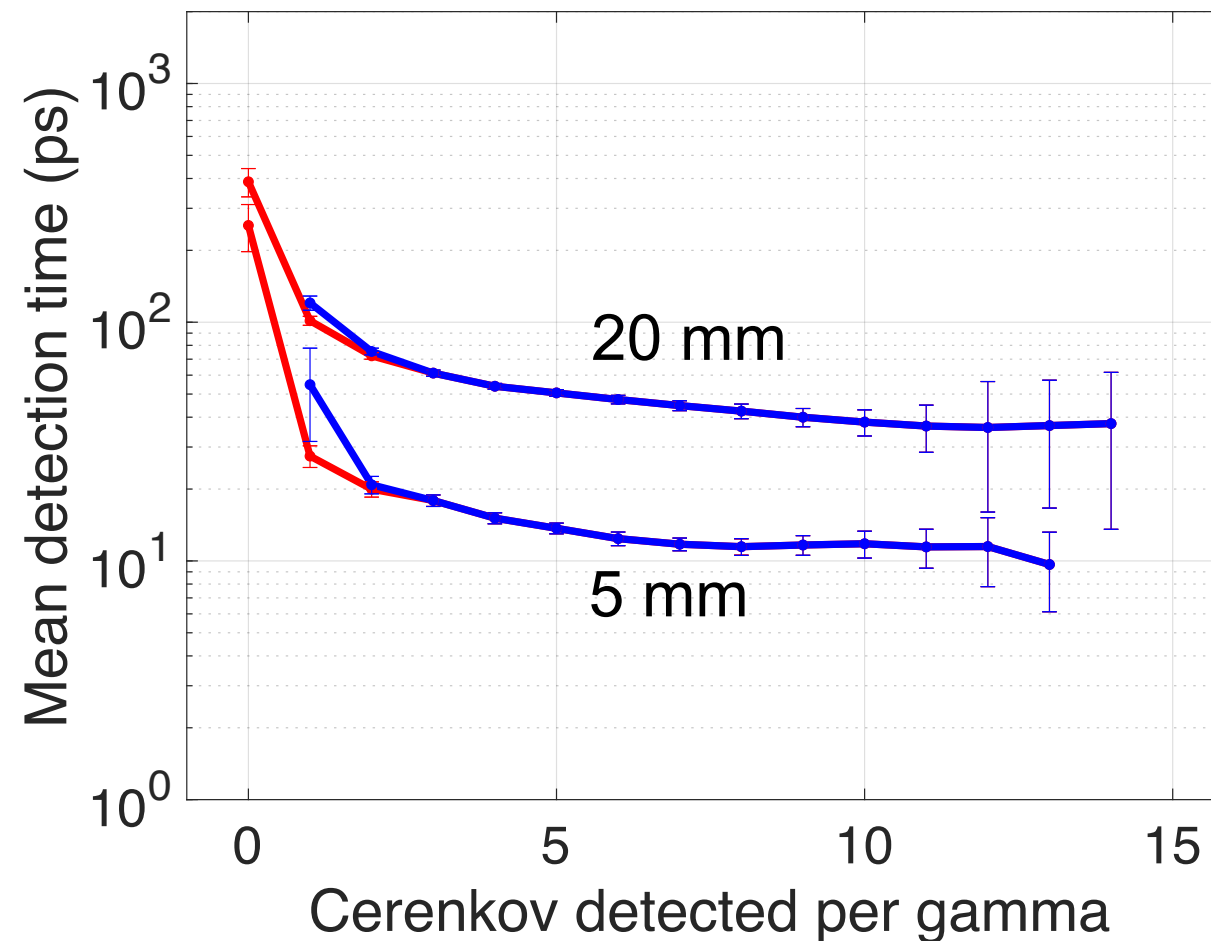**(C)****6 x 6**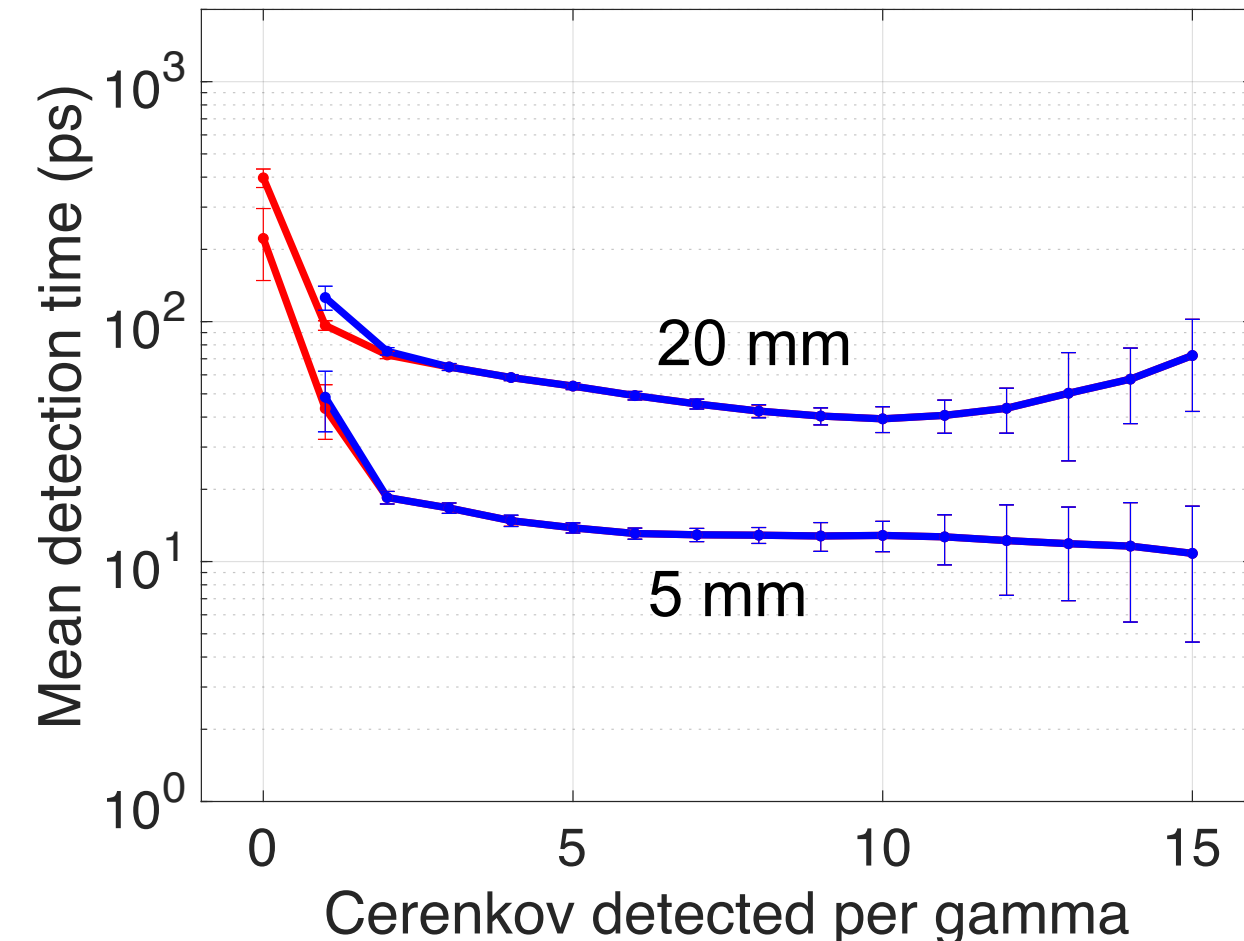**2 x 2**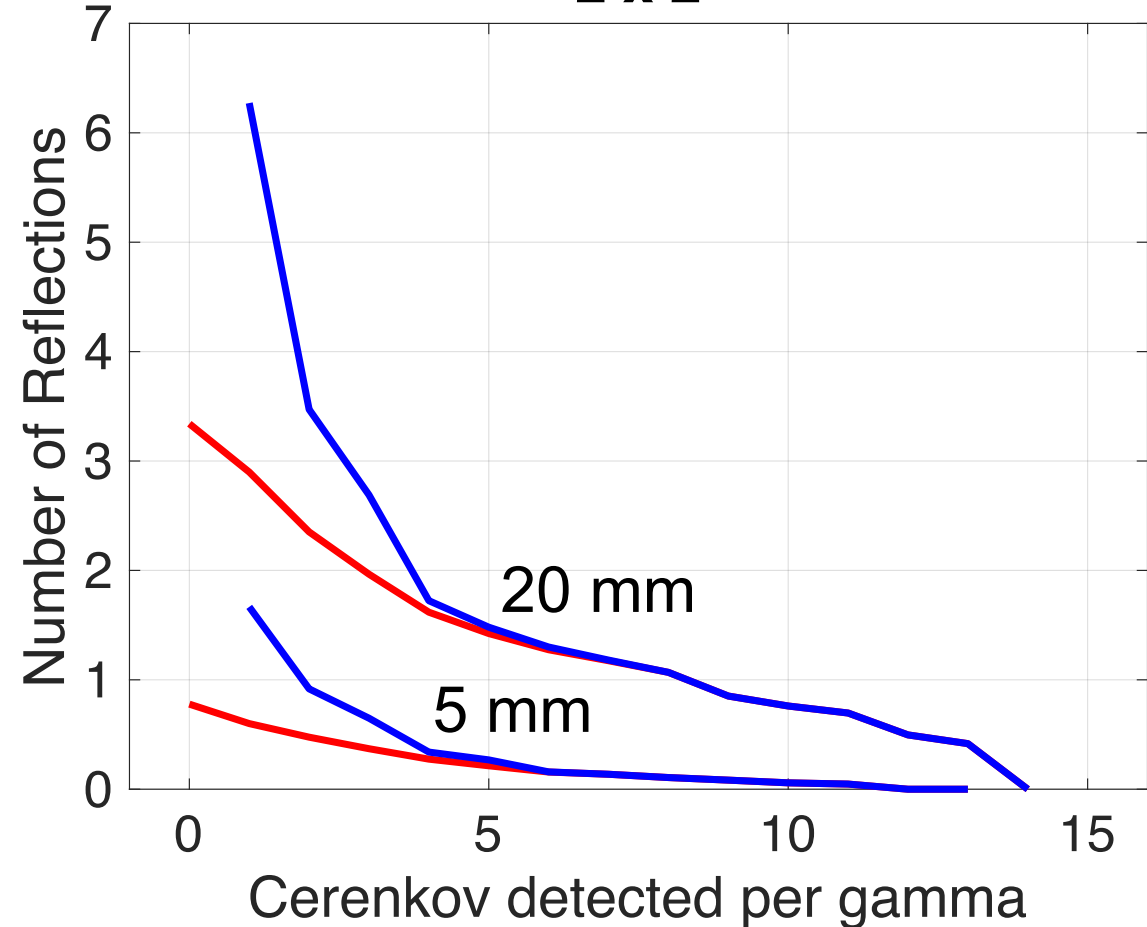**3 x 3**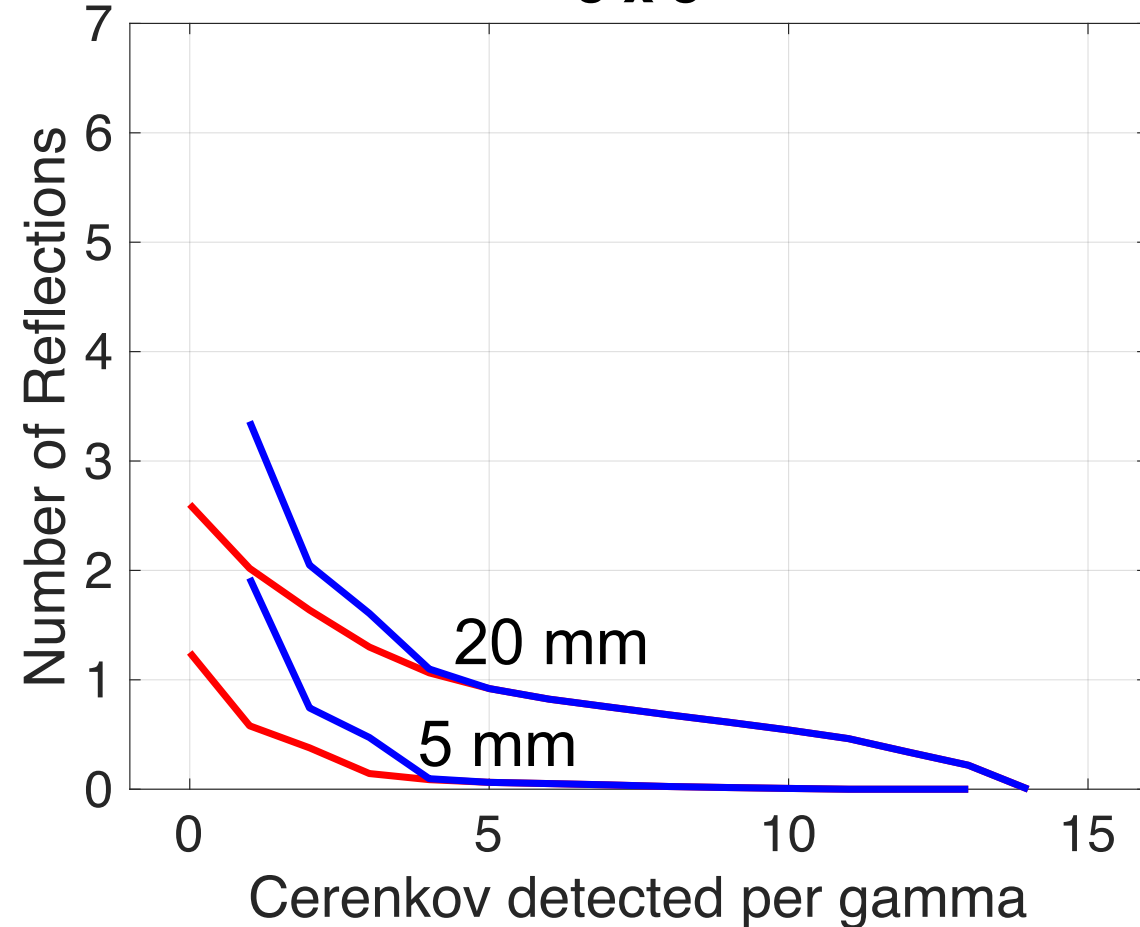**6 x 6**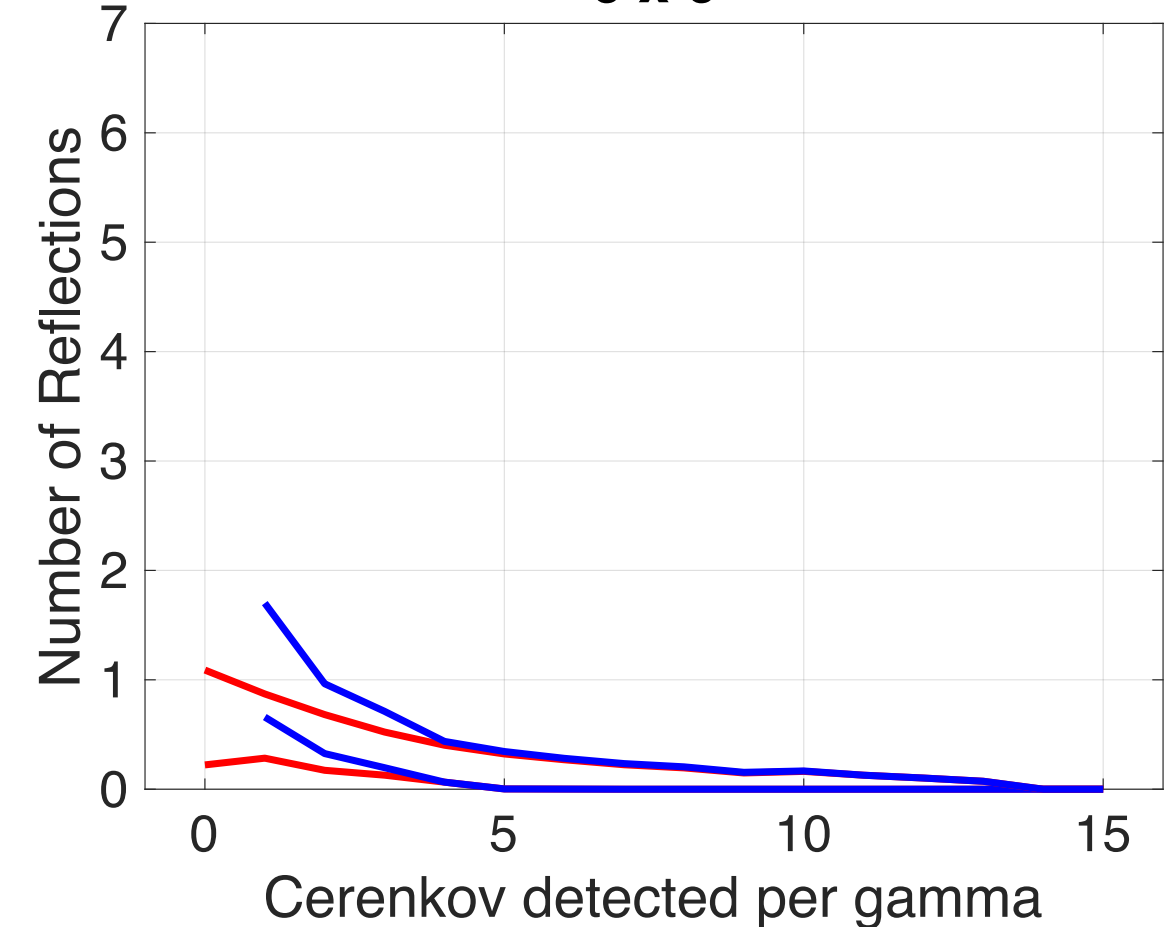**2 x 2**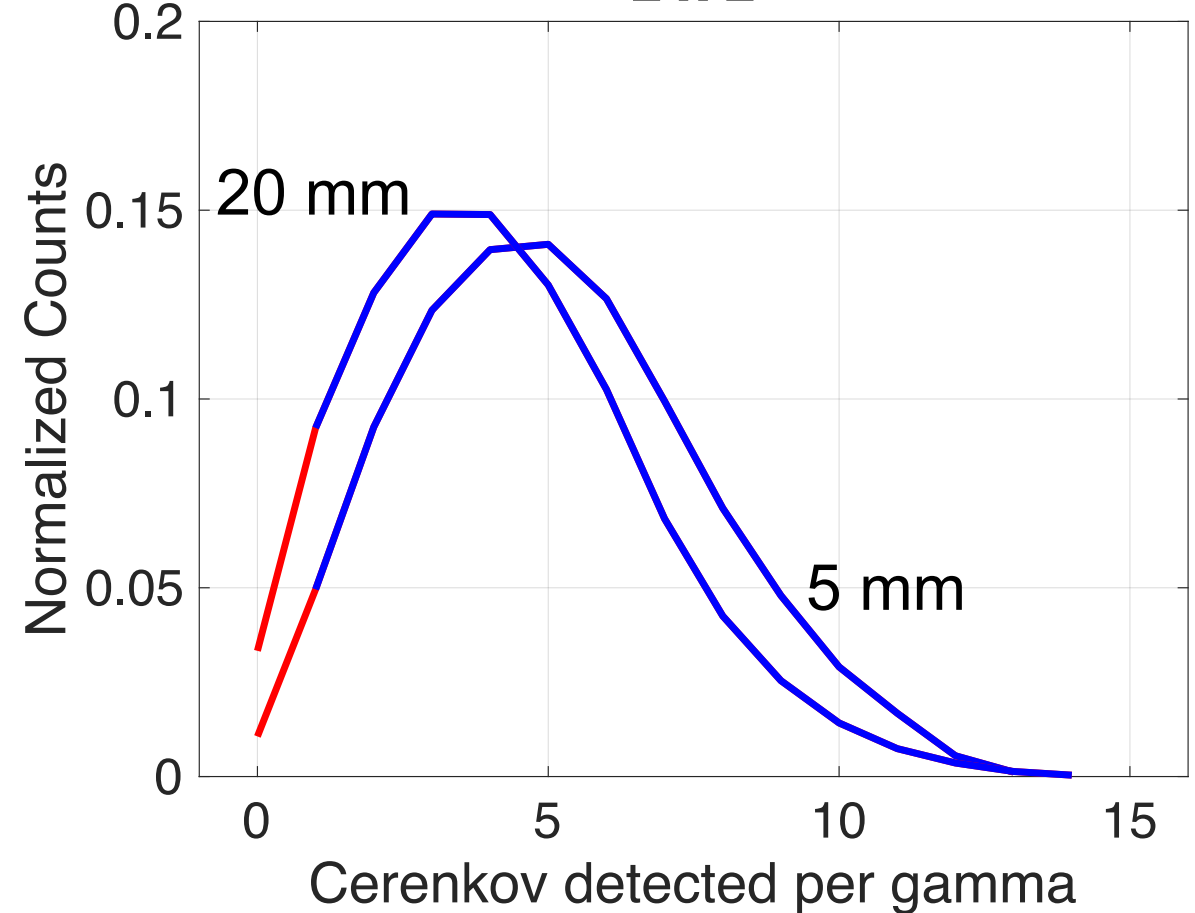**3 x 3**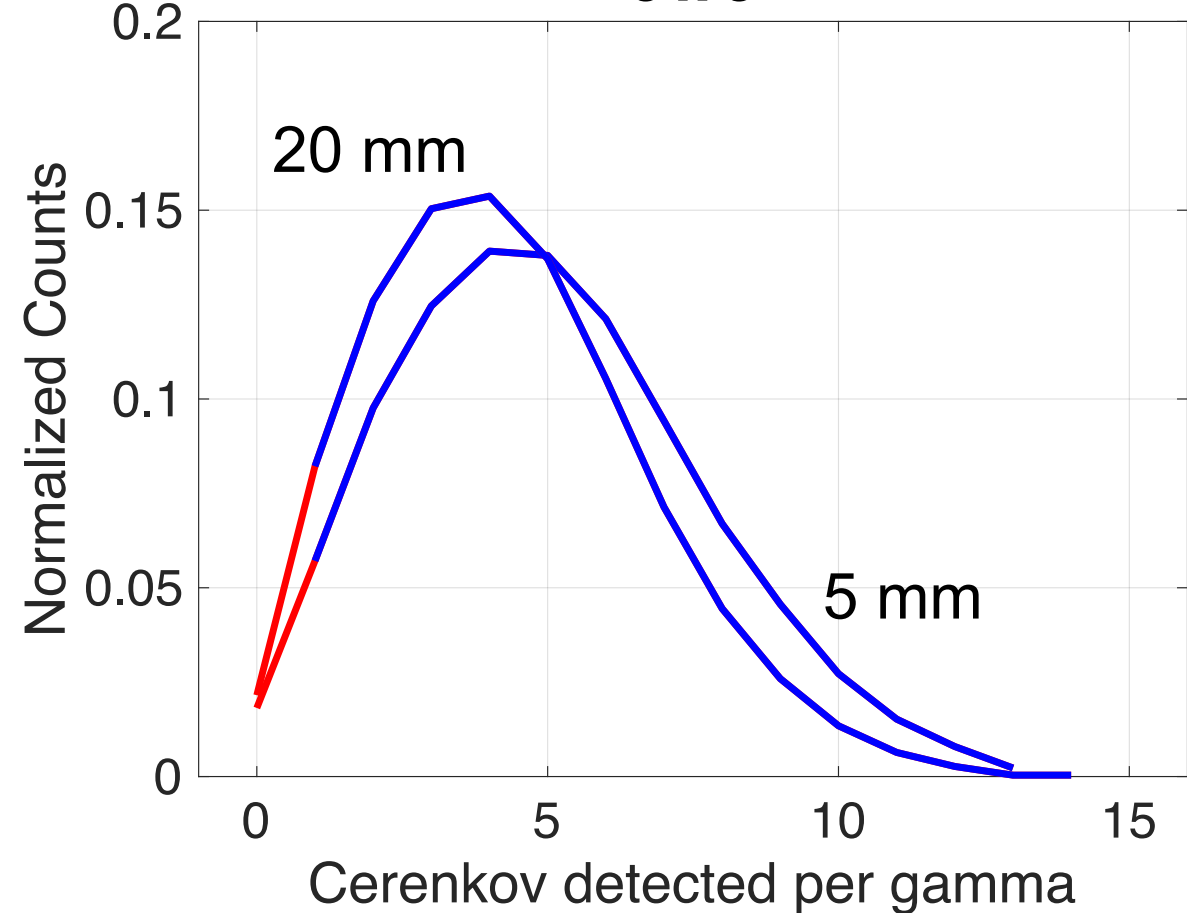**6 x 6**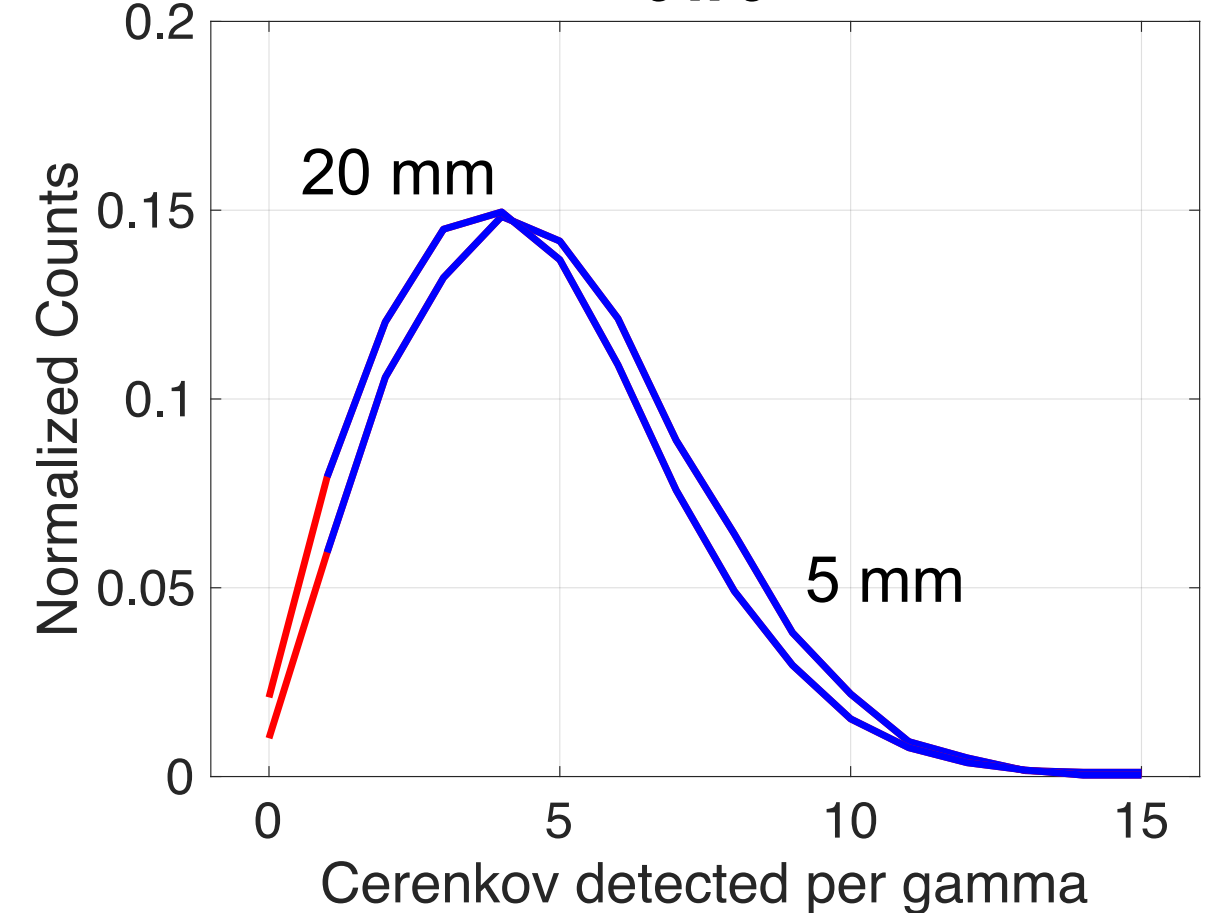

— First detected optical photon

— First detected Cerenkov photon

Supplement: Figure S1 [file NIHMS2002029-supplement-Figure_S1.pdf]

**(A)**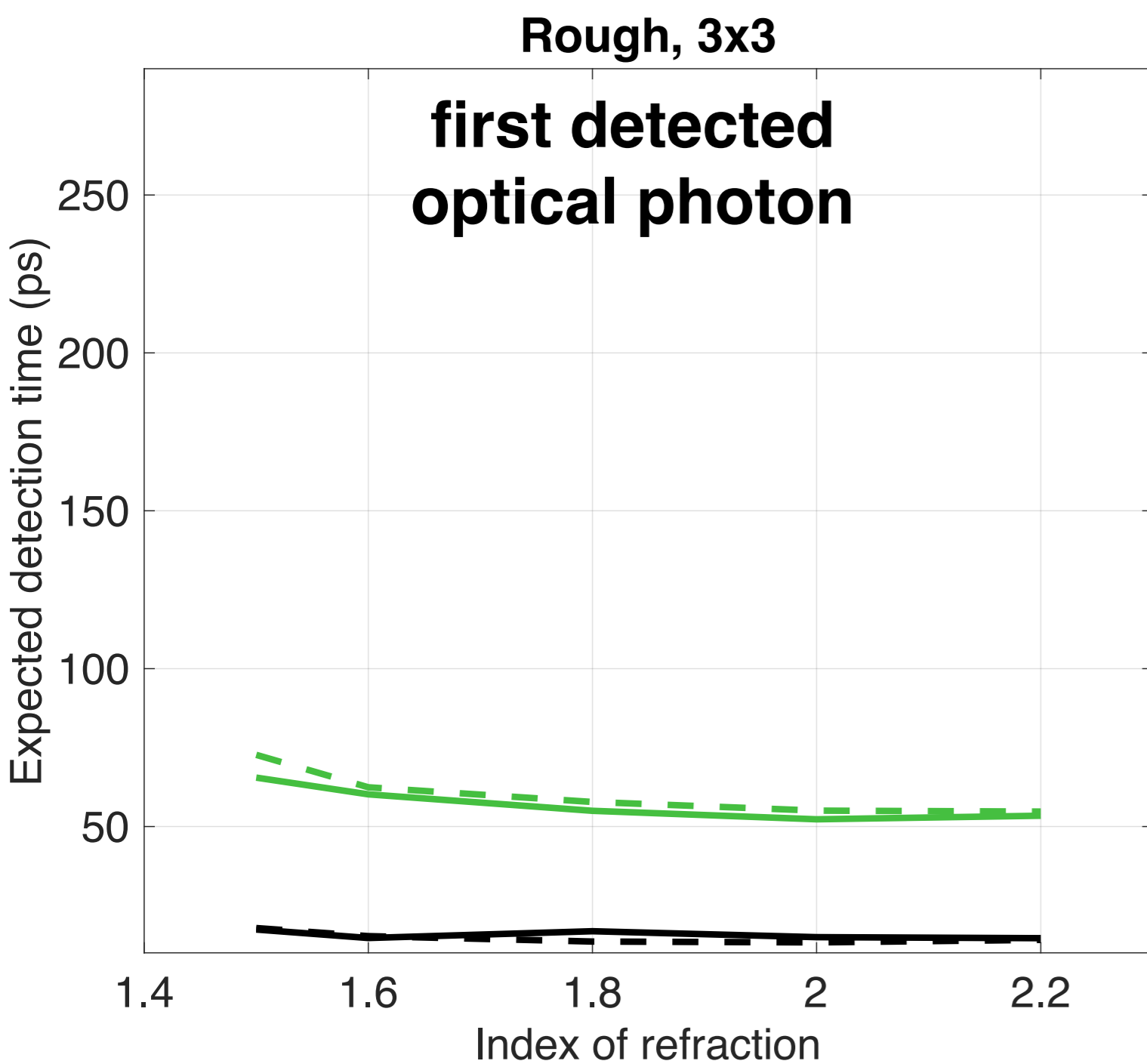**(B)**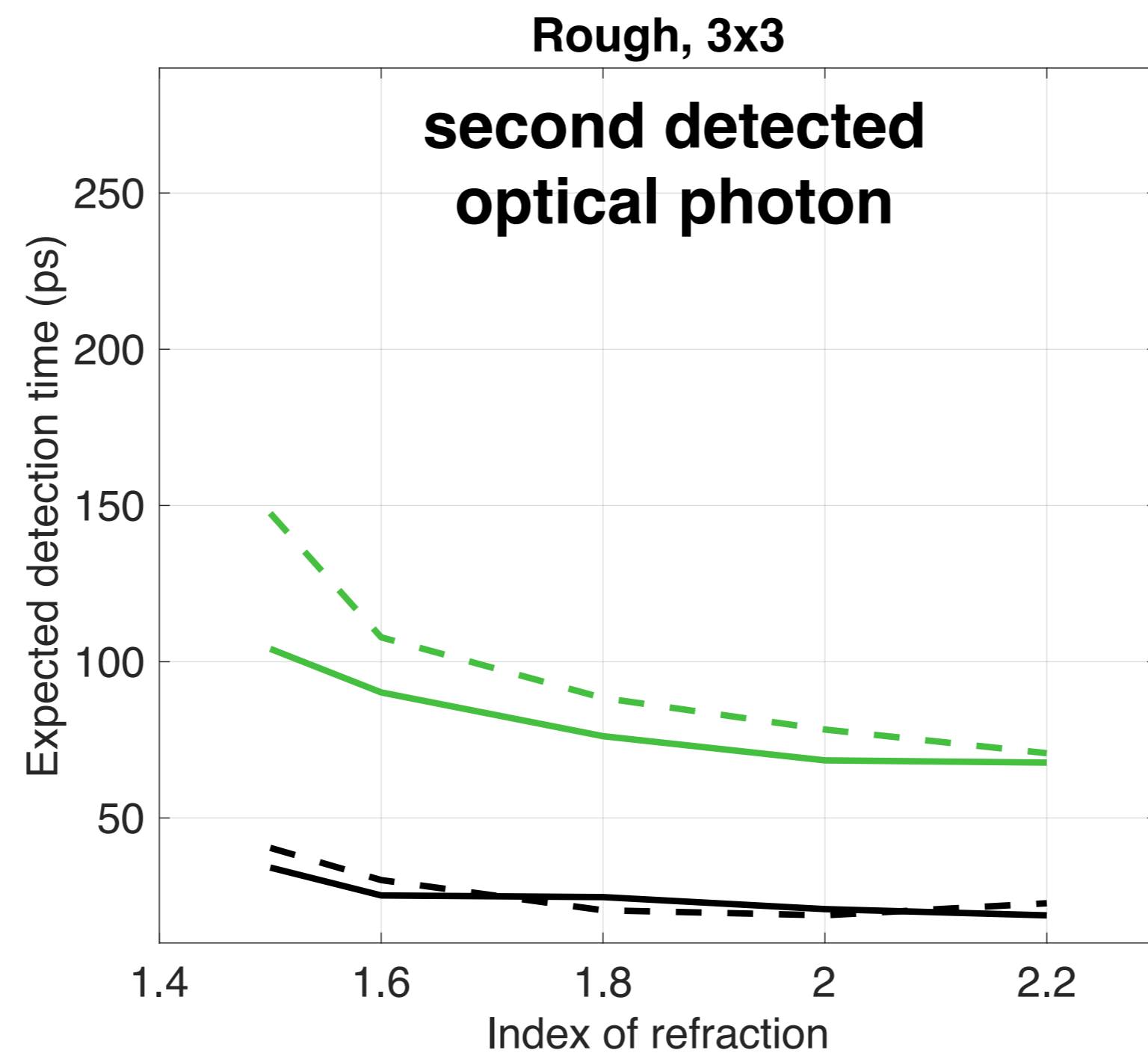**(C)**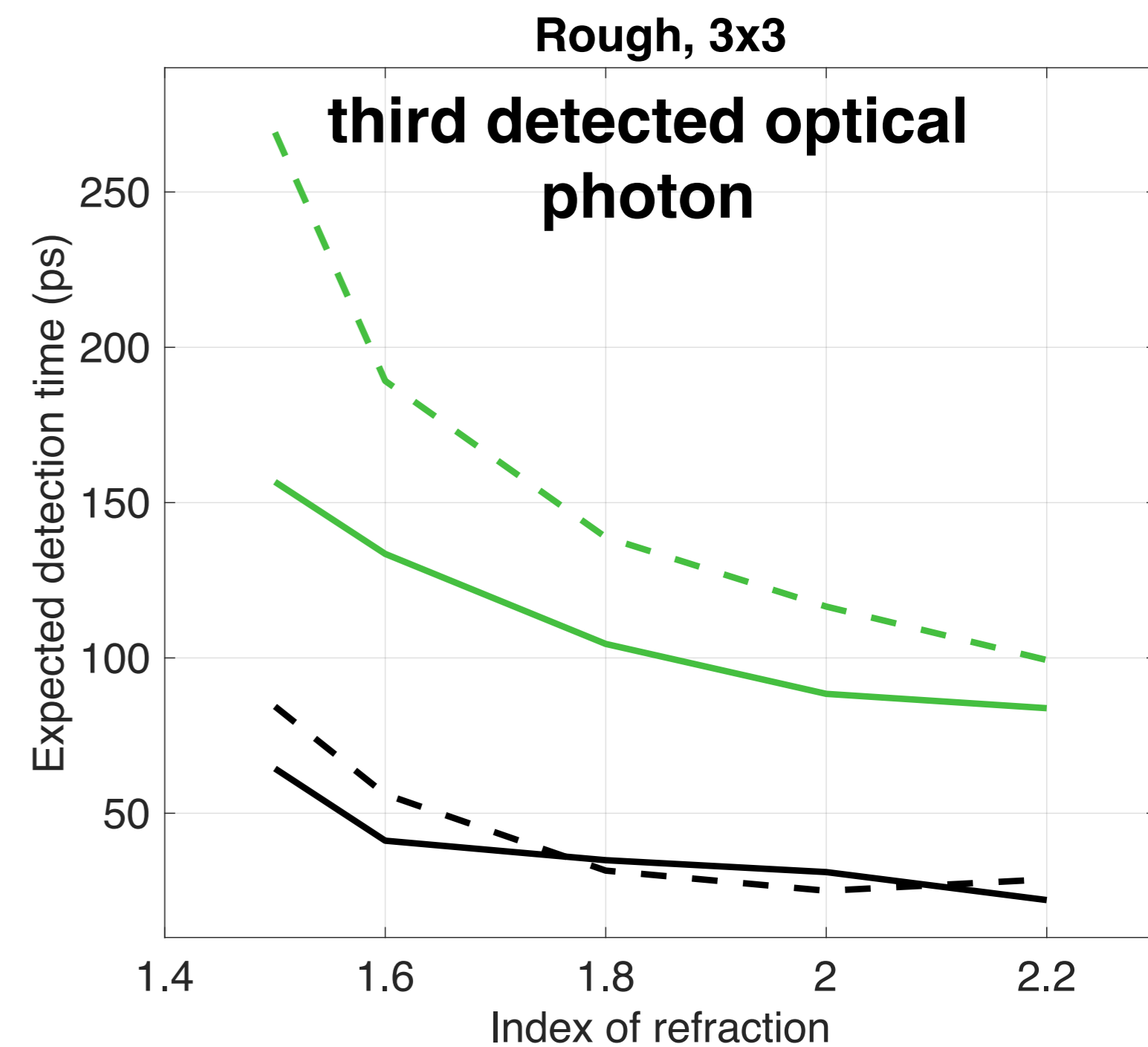

— 5 mm, with reflector      - - - 5 mm, with reflector  
— 20 mm, with reflector      - - - 20 mm, with reflector

Supplement: Figure S10 [file NIHMS2002029-supplement-Figure_S10.pdf]
