# Supplementary material for: Effect of crystal-photodetector interface extraction efficiency on Cerenkov photons’ detection time
Source: Front Phys. Author manuscript; Available in PMC 2024 Dec 23. (PMC11666256; doi:10.3389/fphy.2022.1028293)
Supplement: Figure S2 [file NIHMS2002029-supplement-Figure_S2.pdf]

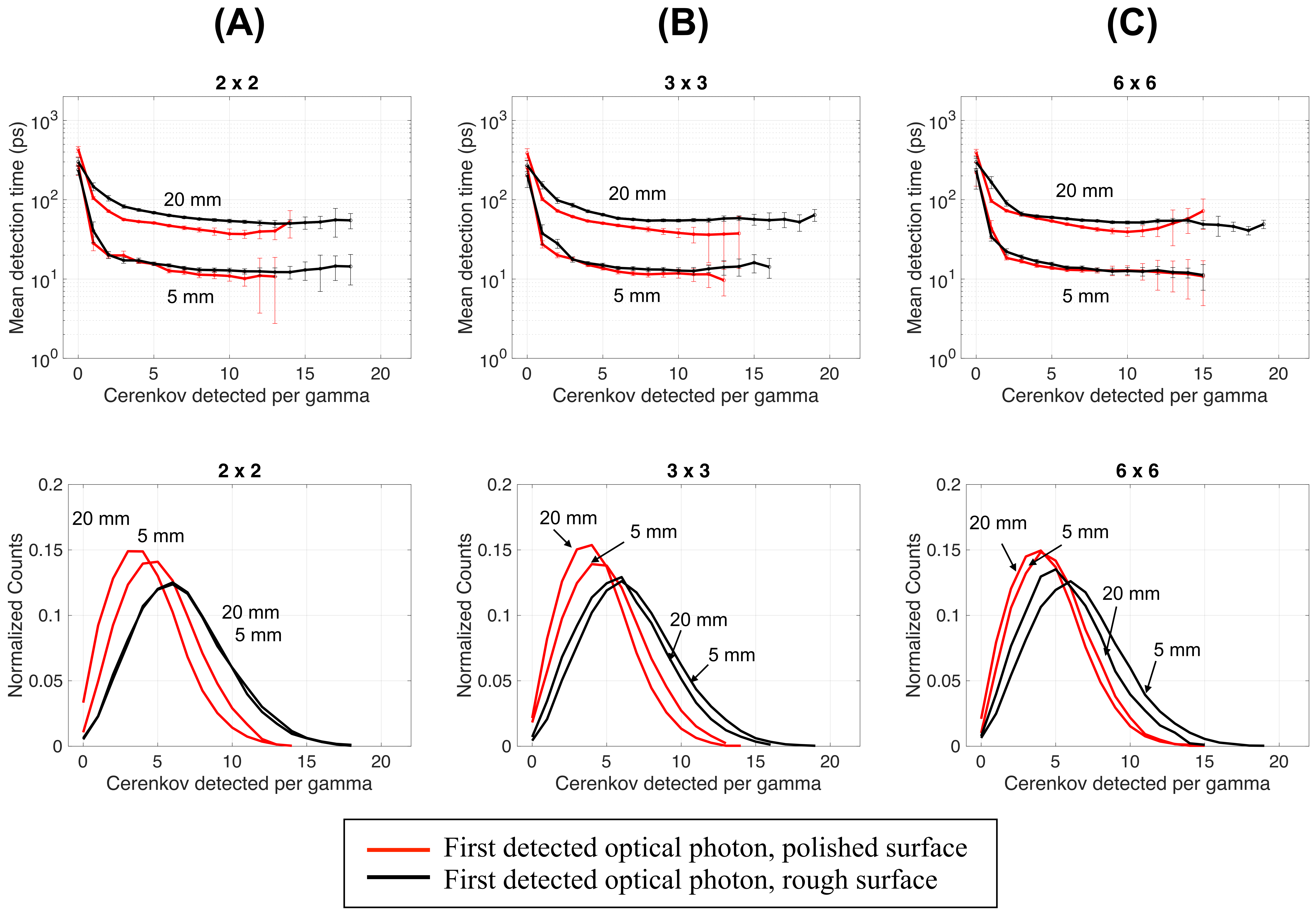

(A)
(B)
(C)
2 x 2
3 x 3
6 x 6
Mean detection time (ps)
20 mm
5 mm
Cerenkov detected per gamma
Normalized Counts
First detected optical photon, polished surface
First detected optical photon, rough surface
