# Supplementary material for: Effect of crystal-photodetector interface extraction efficiency on Cerenkov photons’ detection time
Source: Front Phys. Author manuscript; Available in PMC 2024 Dec 23. (PMC11666256; doi:10.3389/fphy.2022.1028293)
Supplement: Figure S5 [file NIHMS2002029-supplement-Figure_S5.pdf]

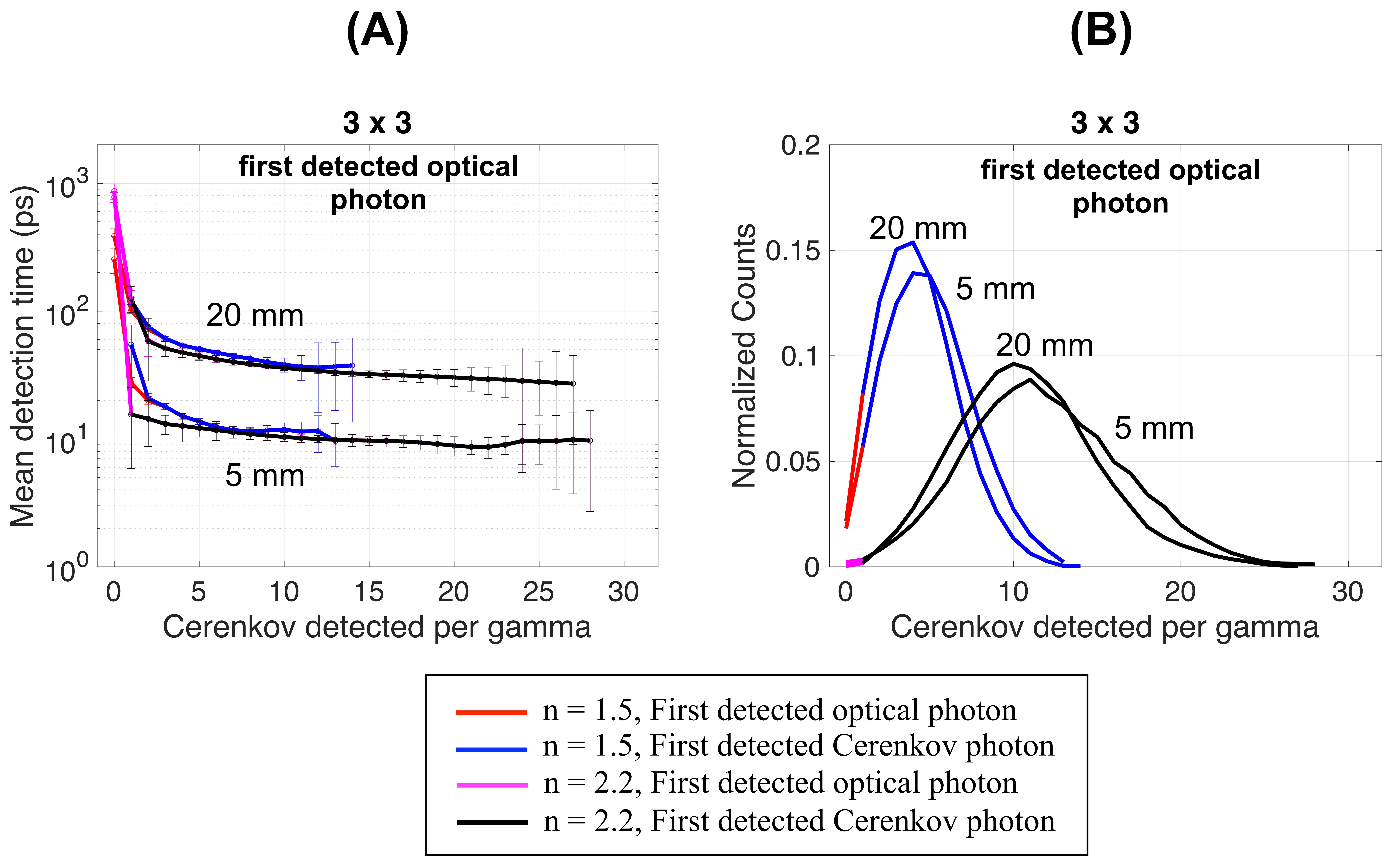

(A)
3 x 3
first detected optical photon
20 mm
5 mm
Mean detection time (ps)
Cerenkov detected per gamma
(B)
3 x 3
first detected optical photon
20 mm
5 mm
20 mm
5 mm
Normalized Counts
Cerenkov detected per gamma
n = 1.5, First detected optical photon
n = 1.5, First detected Cerenkov photon
n = 2.2, First detected optical photon
n = 2.2, First detected Cerenkov photon
