# Supplementary material for: Effect of crystal-photodetector interface extraction efficiency on Cerenkov photons’ detection time
Source: Front Phys. Author manuscript; Available in PMC 2024 Dec 23. (PMC11666256; doi:10.3389/fphy.2022.1028293)
Supplement: Figure S6 [file NIHMS2002029-supplement-Figure_S6.pdf]

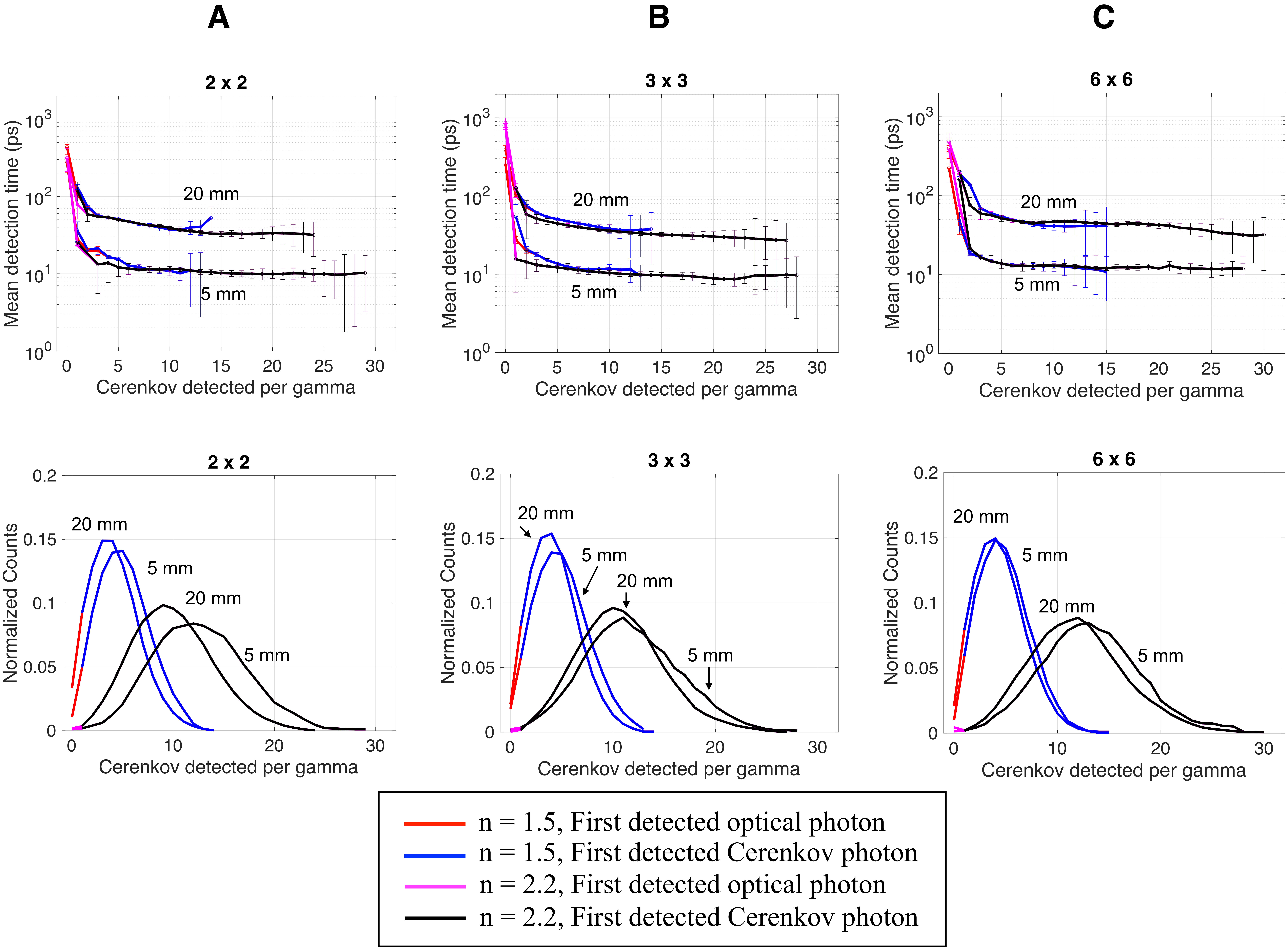
A
B
C
2 x 2
3 x 3
6 x 6
Mean detection time (ps)
Cerenkov detected per gamma
20 mm
5 mm
Normalized Counts
n = 1.5, First detected optical photon
n = 1.5, First detected Cerenkov photon
n = 2.2, First detected optical photon
n = 2.2, First detected Cerenkov photon
