# Supplementary material for: Effect of crystal-photodetector interface extraction efficiency on Cerenkov photons’ detection time
Source: Front Phys. Author manuscript; Available in PMC 2024 Dec 23. (PMC11666256; doi:10.3389/fphy.2022.1028293)
Supplement: Figure S8 [file NIHMS2002029-supplement-Figure_S8.pdf]

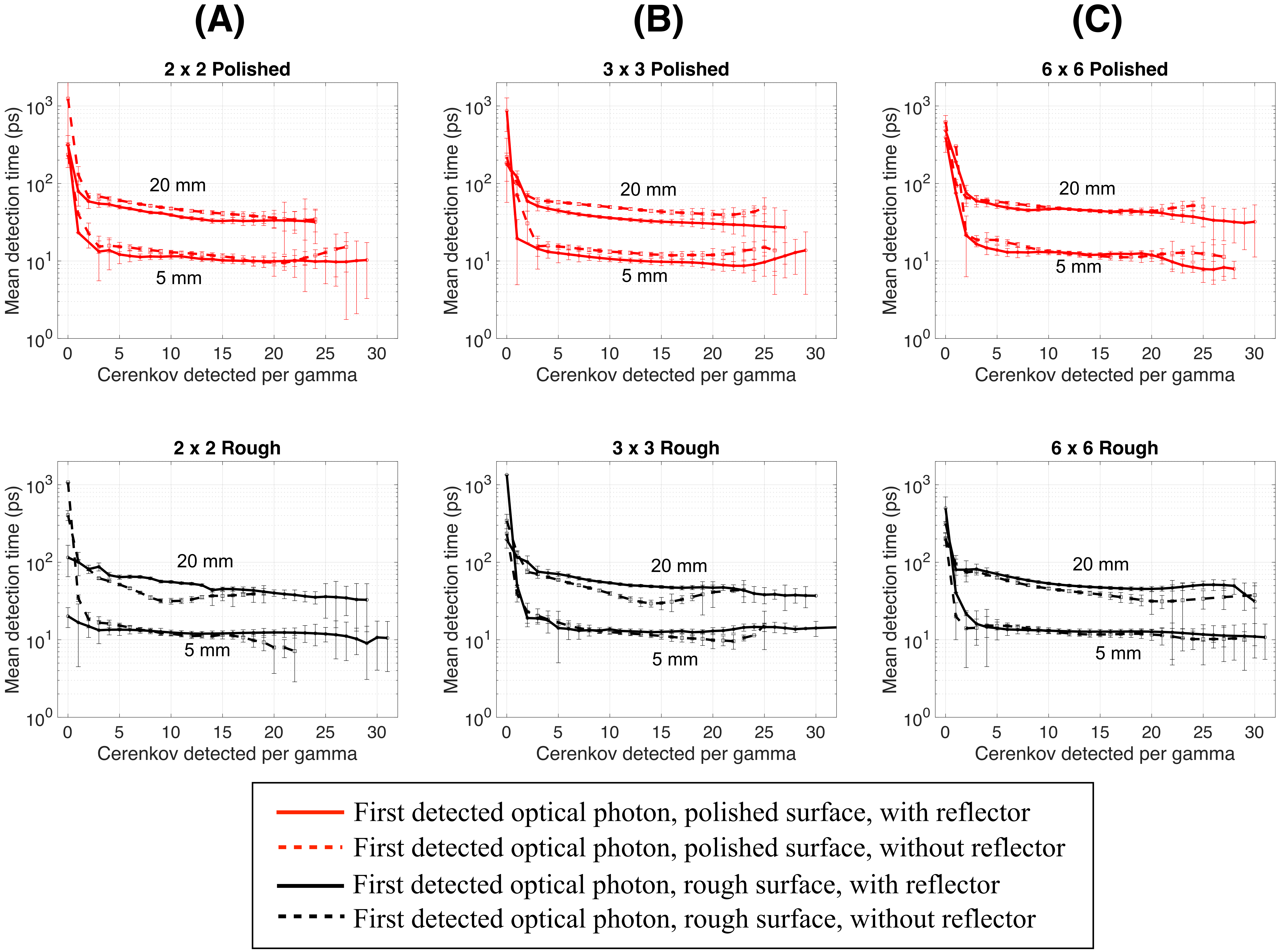

(A)
(B)
(C)
2 x 2 Polished
3 x 3 Polished
6 x 6 Polished
2 x 2 Rough
3 x 3 Rough
6 x 6 Rough
Mean detection time (ps)
Cerenkov detected per gamma
20 mm
5 mm
First detected optical photon, polished surface, with reflector
First detected optical photon, polished surface, without reflector
First detected optical photon, rough surface, with reflector
First detected optical photon, rough surface, without reflector
