# Supplementary material for: Effect of crystal-photodetector interface extraction efficiency on Cerenkov photons’ detection time
Source: Front Phys. Author manuscript; Available in PMC 2024 Dec 23. (PMC11666256; doi:10.3389/fphy.2022.1028293)
Supplement: Figure S9 [file NIHMS2002029-supplement-Figure_S9.pdf]

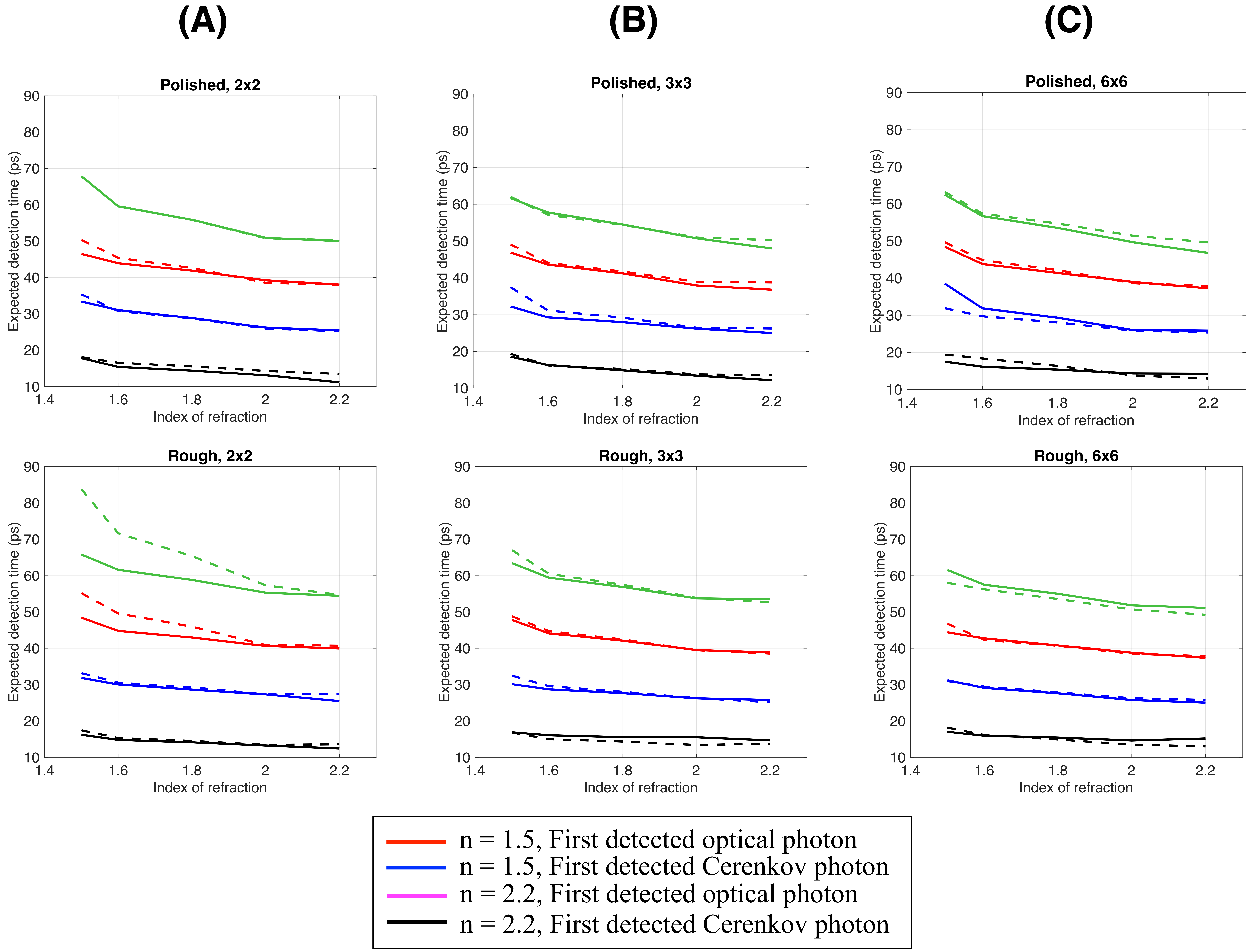
(A)
(B)
(C)
Polished, 2x2
Polished, 3x3
Polished, 6x6
Rough, 2x2
Rough, 3x3
Rough, 6x6
Expected detection time (ps)
Index of refraction
n = 1.5, First detected optical photon
n = 1.5, First detected Cerenkov photon
n = 2.2, First detected optical photon
n = 2.2, First detected Cerenkov photon
